# An *in silico* down-scaling approach uncovers novel constituents of the *Plasmodium*-containing vacuole

**DOI:** 10.1038/s41598-018-32471-6

**Published:** 2018-09-19

**Authors:** Joachim Michael Matz, Kai Matuschewski

**Affiliations:** 1 Department of Molecular Parasitology, Institute of Biology, Humboldt University, 10115 Berlin, Germany; 2 Parasitology Unit, Max Planck Institute for Infection Biology, 10117 Berlin, Germany

## Abstract

During blood stage development the malaria parasite resides in a membrane-bound compartment, termed the parasitophorous vacuole (PV). The reasons for this intravacuolar life style and the molecular functions of this parasite-specific compartment remain poorly defined, which is mainly due to our limited knowledge about the molecular make-up of this unique niche. We used an *in silico* down-scaling approach to select for *Plasmodium*-specific candidates that harbour signatures of PV residency. Live co-localisation of five endogenously tagged proteins confirmed expression in the PV of *Plasmodium berghei* blood and liver stages. ER retention was ruled out by addition of the respective carboxyterminal tetrapeptides to a secreted reporter protein. Although all five PV proteins are highly expressed, four proved to be dispensable for parasite development in the mammalian and mosquito host, as revealed by targeted gene deletion. In good agreement with their redundant roles, the knockout parasites displayed no detectable deficiencies in protein export, sequestration, or PV morphology. Together, our approach improved the catalogue of the *Plasmodium* PV proteome and provides experimental genetics evidence for functional redundancy of several PV proteins.

## Introduction

Throughout blood infection, the malaria parasite resides inside a membrane-bound compartment, termed the parasitophorous vacuole (PV). The PV is initially formed from an invagination of the host cell during invasion and is, therefore, originally derived from the plasma membrane of the infected RBC (iRBC)^1–3^. During blood stage development, the PV grows in size and complexity and allows for extensive refurbishment of the infected host cell. The trafficking of membranes and proteins through the PV into the iRBC is a major parasite-induced mechanism to hijack the cellular functions of the erythrocyte^4,5^. During protein export into the host cell, the PV membrane (PVM) selectively permits the passage of some proteins and ensures the retention of others, further highlighting its gateway function in host cell refurbishment.

A wealth of knowledge has been gathered about exported proteins and their roles in parasite development and virulence^6^. However, the fundamental purpose of their gateway, the PV, remains unknown. Most functions attributed to the PV, like nutrient acquisition and protein translocation^7,8^, are merely complex mechanisms of coping with the existence of such a restrictive compartment. While membranous envelopes serve as protective hiding spaces for many intracellular pathogens, the reasons for *Plasmodium* to stay inside a vacuole remain elusive. The RBC is a terminally differentiated cell, which, in contrast to other cell types, does not possess the capacity to detect or destroy the pathogen. Therefore, it seems puzzling, why the parasite would limit host cell access, while growing in such a safe haven. Indeed, the closely related piroplasmid parasites *Babesia* and *Theileria* are known to initially form a PV. However, upon successful invasion, both parasites degrade their temporal envelope and thrive in the RBC cytoplasm^9,10^. Nonetheless, *Babesia* parasites have been shown to reproduce most aspects of *Plasmodium* biology with regards to host cell remodelling and pathology despite the lack of a PV^11^. Therefore, we are left to speculate what the ultimate functions of the plasmodial PV are, other than compensating for the inconvenience of its very existence.

One of the main reasons for our limited understanding of the PV is the lack of comprehensive proteomic data. Recent efforts using proximity-based biotinylation or label-free subcellular fractionation have uncovered several novel PV constituents^12–14^. However, the predictive accuracy of these approaches was unsatisfactory, indicating that the majority of PV proteins remain unrecognised.

In this work, we describe the *in silico* identification, validation and functional investigation of novel PV proteins by experimental genetics in the murine malaria model parasite *Plasmodium berghei*.

## Results

### *In silico* identification of PV candidates

**Figure 1:**
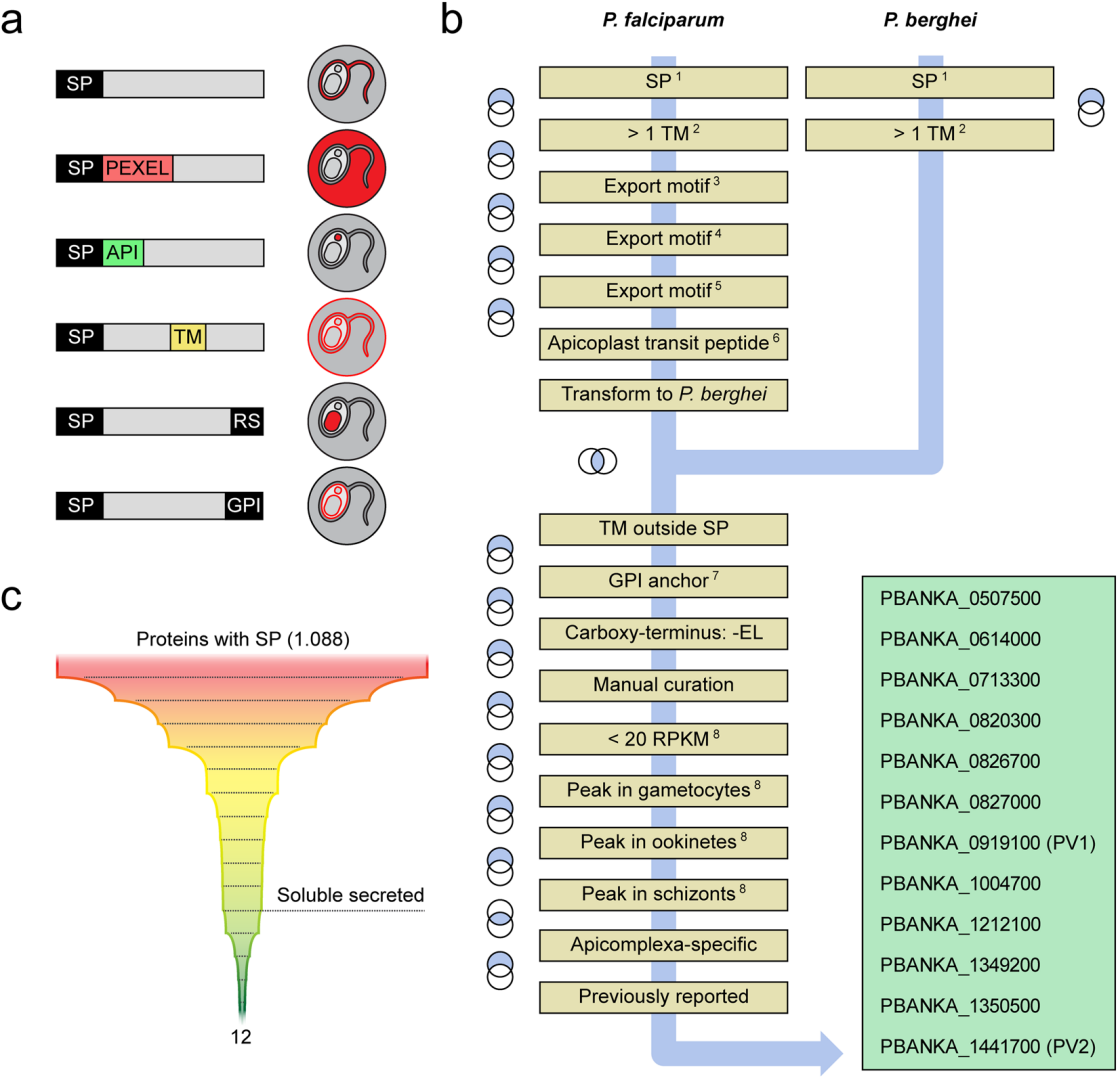
*In silico* identification of *Plasmodium* PV protein candidates. (**a**) *Plasmodium* protein targeting in infected erythrocytes *via* the secretory pathway. Depicted are schematic representations of proteins with different targeting information and their expected localisation patterns during blood stage development. SP, signal peptide; PEXEL, *Plasmodium* export element; API, apicoplast transit peptide; TM, transmembrane domain; RS, endoplasmic reticulum retention signal; GPI, glycosylphosphatidylinositol anchor. (**b**) Algorithm for the *in silico* identification of PV candidates. Shown is a schematic representation of the selection procedure. The blue arrow denotes the sequence of events. Individual steps are shown in yellow. Venn diagrams indicate whether the relative complement or the intersection of two steps was used. SP-containing *Plasmodium* proteins were selected in *P. falciparum* and *P. berghei*. Proteins containing additional targeting information were removed sequentially. Proteins with annotated functions outside the PV, *e.g*. mitochondrial or nuclear proteins, were removed manually. Note that several selected proteins were assigned a predicted transmembrane (TM) domain at the amino-terminus due to the hydrophobicity of the SP. Next, genes showing very weak expression in asexual blood stages or peak expression in schizonts, gametocytes and/or ookinetes were excluded. Finally, previously neglected proteins specific to the Apicomplexa or to the genus *Plasmodium* were selected, including PV1 and 2, which served as positive controls for experimental validation. Accession codes of the final *P. berghei* PV candidates are shown in the green box. 1, Predicted with SignalP^41^; 2, predicted with TMHMM^42^; 3, *Plasmodium* export element^34^; 4, host targeting motif ^33^; 5, predicted with ExportPred^35^. 6, predicted with PlasmoAP^43^; 7, predicted with big-Pi^44^; 8, transcript abundance determined by RNA sequencing^16^. (**c**) Representation of the applied search algorithm and the associated numbers of proteins.

Proteins are targeted to the PV by means of default protein secretion, which is initiated by the recognition and cleavage of an amino-terminal signal peptide (SP)^15^. To identify novel PV candidates, we searched the *Plasmodium* genome database (*PlasmoDB)* for SP-containing proteins and sequentially removed proteins containing predicted transmembrane domains, export motifs, apicoplast targeting peptides, endoplasmic reticulum (ER) retention signals and GPI anchors, which could potentially redirect the protein to other locations (Fig. 1a,b). In order to identify proteins which carry out functions inside the PV during asexual parasite growth, we removed genes showing marked peak transcription in either schizonts, gametocytes or ookinetes, thereby eliminating genes involved in motility, invasion and host transition^16^. Established PV constituents, such as the components of the *Plasmodium* translocon of exported proteins (PTEX), and other proteins with already reported localisation, were excluded. The known vacuolar proteins PV1^17^ and PV2^18^ were also recovered by the search algorithm. We included both proteins as positive controls, resulting in 12 apicomplexan-specific PV candidates (Fig. 1b,c).

### Validation of PV targeting

**Figure 2:**
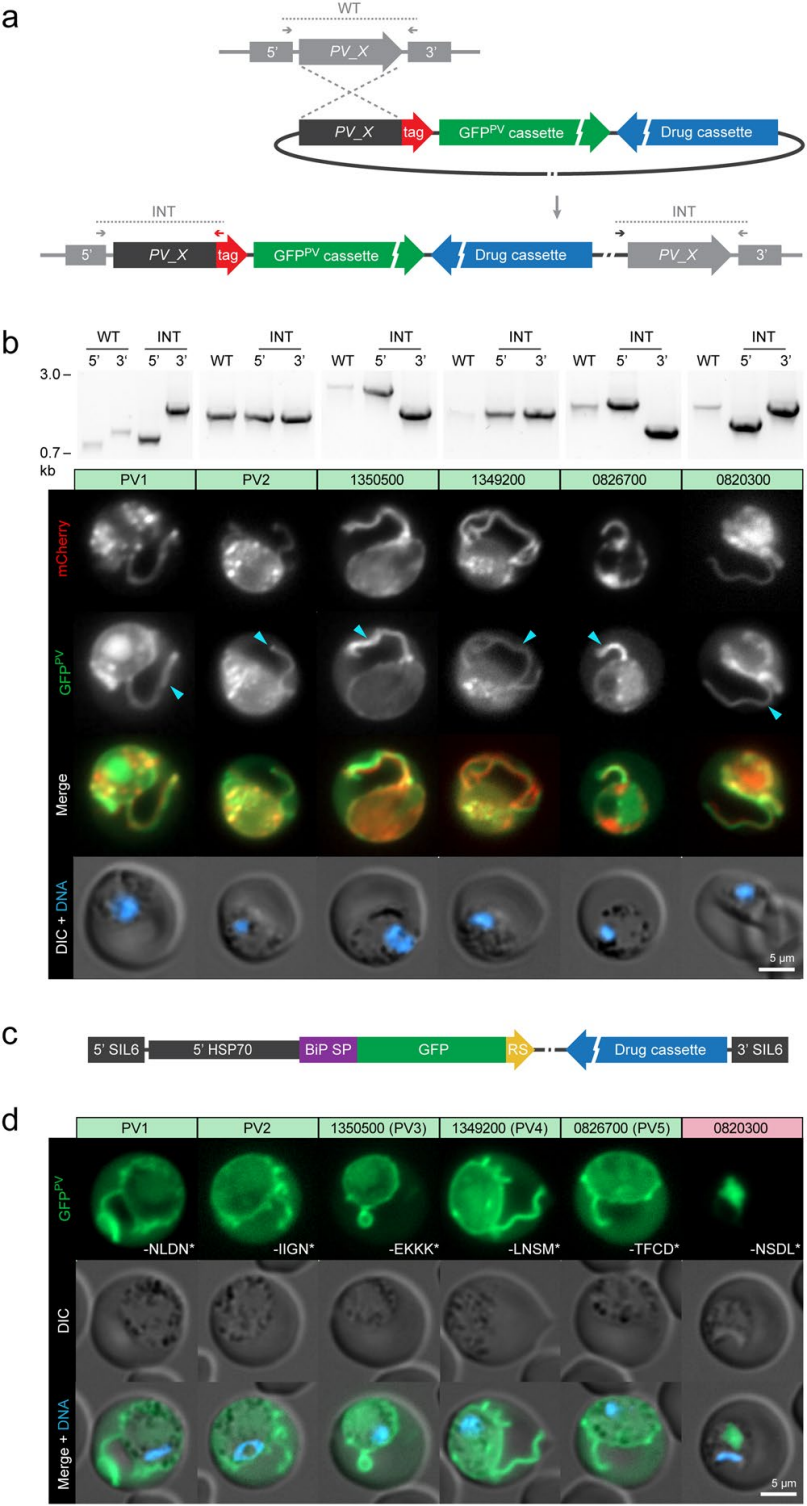
Validation of novel PV proteins. (**a**) Strategy for the generation of transgenic parasite lines expressing endogenous PV proteins (*PV_X*) fused to mCherry-3xMyc (tag) by single homologous integration. In addition recombinant parasites contain the drug-selectable hDHFR-yFcu cassette (drug cassette) and the GFP^PV^ cassette^19^. Wild-type (WT) and integration-specific (INT) primer combinations (Supplementary Table S1) are indicated by arrows and expected fragments by dotted lines. Note that PV1 and PBANKA_1212100 were tagged using another strategy based on double homologous recombination (see Supplementary Fig. S1). (**b**) Live fluorescence microscopy of selected PV candidates. Numbers indicate gene accession codes (without the ‘PBANKA_’ prefix). Shown are transgenic *P. berghei* blood stage parasites expressing the endogenous candidate genes fused to mCherry-3xMyc (red, top) and the marker protein GFP^PV^ (green, lower top) as well as a merge of both fluorescent protein signals (upper bottom) and a merge of differential interference contrast images (DIC) with Hoechst 33342 nuclear dye (DNA, blue, bottom). The known PV proteins PV1 and PV2 are included as positive controls. , PV protrusions. Candidates showing no PV localisation are depicted in Supplementary Fig. S1. Wild-type (WT) and integration-specific (INT) diagnostic PCRs of the parental transgenic parasite lines, as indicated in a and Supplementary Fig. S1, are shown above and indicate proper genomic integration of the targeting constructs. For full size images of DNA gels, see Supplementary Fig. S5. (**c,d**) Assessment of ER retention. (**c**) Schematic representation of the vectors used for ER retention testing. The GFP^PV^ cassette, consisting of the signal peptide of BiP (purple) fused to GFP (green), was appended with the last four amino acids of the PV candidates (yellow) and expressed from the silent intergenic locus on chromosome 6 (SIL6) under the control of the *HSP70* promoter. (**d**) Live fluorescence microscopy of transgenic *P. berghei* parasites expressing ER retention testing constructs. Shown are the GFP signal (green, top), differential interference contrast images (DIC, middle), and a merge with Hoechst 33342 nuclear dye (DNA, blue, bottom). The white labelling indicates the last four amino acids of the PV candidates.

In order to test whether the selected candidates indeed localise to the PV we generated transgenic *P. berghei* parasites that express the endogenous candidate genes fused to a fluorescent mCherry-3xMyc tag (Fig. 2a and Supplementary Fig. S1). In addition, the transfection vectors contained the PV marker cassette GFP^PV^ for live protein co-localisation^19^. Correct genomic integration of the DNA constructs was confirmed by diagnostic PCR (Fig. 2b and Supplementary Fig. S1).

Upon inspection of peripheral blood, we could confirm that PV1 and PV2 localise to the blood stage PV as indicated by their residence in elongated GFP^PV^-positive membrane protrusions (Fig. 2b)^19^. As shown previously, a fraction of the GFP^PV^ marker leaks into the host cell cytoplasm due to its very high level of expression^19^. We found that 5 of the selected candidates did not localise to the PV (Supplementary Fig. S1). Surprisingly, 3 selected candidates exhibited a cytoplasmic distribution highlighting the limitations of *in silico* SP-prediction: PBANKA_0713300 encodes a member of the histidine triad superfamily of nucleotide hydrolases and nucleotide transferases; PBANKA_1004700 contains a signature methyltransferase domain; PBANKA_1212100 encodes a hypothetical protein of unknown function. One candidate, PBANKA_0614000, encoding a hypothetical protein, localised to a distinct structure, suggestive of a parasite organelle of yet unknown identity. PBANKA_0827000, which encodes a short hydrophobic polypeptide of 51 amino acid residues, appeared to be associated with the parasite surface, but was excluded from PV tubules. A sixth candidate, PBANKA_0507500 encoding a hydrophobic polypeptide of 79 amino acid residues, remained refractory to endogenous tagging, most likely due to an interference of the fluorescent tag with essential protein functions (Supplementary Fig. S1).

Importantly, four previously unrecognised candidates showed localisation to tubular PV protrusions, which were indistinguishable from the localisation of PV1 and PV2 (Fig. 2b). To further validate their localisation, we tested whether the fluorescent tag interferes with the recognition of carboxy-terminal ER-retention signals, thereby leading to erroneous secretion into the PV. Although we have excluded proteins with a predicted ER retrieval signal (*i.e*. ending on -EL) during *in silico* analysis (Fig. 1b), deviant amino acid combinations have been reported to also promote ER retention^20^. Thus, we fused the last four amino acids of the PV candidates to GFP^PV^ and assessed the reporter’s localisation during blood stage development (Fig. 2c). All reporters were secreted into the PV, with the exception of the PBANKA_0820300 reporter, which was found to be retained inside the parasite indicative of ER localisation (Fig. 2d). PBANKA_0820300 encodes a putative protein disulfide isomerase and ends on -NSDL, which appears to serve as a cryptic ER retrieval motif. We have thus removed this protein from our selection. In contrast, we could validate the PV localisation of PBANKA_1350500, PBANKA_1349200 and PBANKA_0826700, which we have dubbed PV3, PV4, and PV5, respectively.

### PV1-5 are expressed throughout asexual blood stage development

We then asked whether PV1-5 are also expressed during other stages of the intraerythrocytic life cycle. Thus, we imaged the fluorescently tagged parasite lines during the ring, trophozoite and schizont stages. All five proteins were found to be associated with the parasite periphery and with PV-derived tubules and vesicular structures throughout the entire asexual cycle (Fig. S2). Strikingly, a substantial fraction of PV4 was found in the erythrocyte cytoplasm during ring and early trophozoite stages. This exported protein fraction progressively disappeared during parasite growth and was not detected in mature trophozoites or schizonts. During the schizont stage, all five proteins localised around the formed merozoites and co-localised with the hemozoin crystals of the digestive vacuoles (Fig. S2). This is most likely due to the internalization of PV material during haemoglobin ingestion^21,22^.

### PV1-5 are not associated with the parasite surface

**Figure 3:**
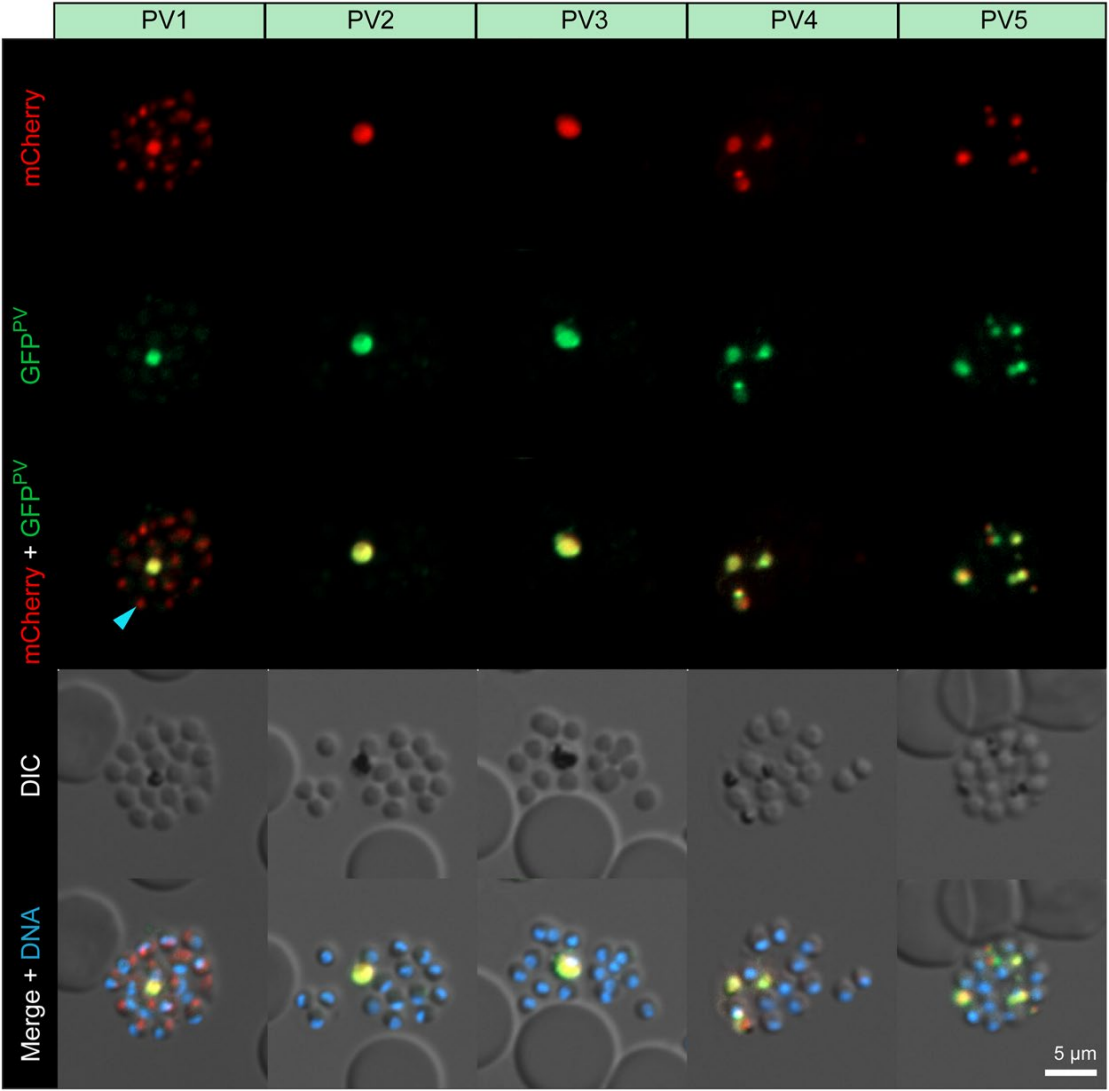
PV1-5 are not associated with the parasite plasma membrane. Shown are transgenic *Plasmodium berghei* blood stage parasites expressing the endogenous PV proteins fused to mCherry-3xMyc (red, top) and GFP^PV^ (green, lower top), a merge of both fluorescent protein signals (middle), differential interference contrast images (DIC, upper bottom) as well as a merge of all signals with Hoechst 33342 nuclear dye (DNA, blue, bottom). Depicted are ruptured schizonts. Note that during this stage the PV proteins predominantly localise to the digestive vacuole and not to the surface of the parasites. , PV1-mCherry-fluorescent dot associated with an emerging merozoite.

We next investigated whether the PV proteins are indeed associated with the vacuolar lumen rather than the parasite surface. To that end, we imaged ruptured schizonts, which have already disintegrated the iRBC membrane and the PV, thus leading to the loss of all soluble PV contents (Fig. 3). Upon merozoite egress all five proteins localised almost exclusively to the digestive vacuoles. Upon inspection of individual emerging merozoites, PV2-5 were hardly detected, whereas PV1 was found to be associated with a distinct intraparasitic structure, possibly reflecting localisation to parasite dense granules, as previously demonstrated for other PV proteins^23^. In no instance were the PV proteins detected on the surface of emerging merozoites further supporting the notion that they are indeed mostly luminal (Fig. 3).

### PV1-5 are expressed during liver infection

**Figure 4:**
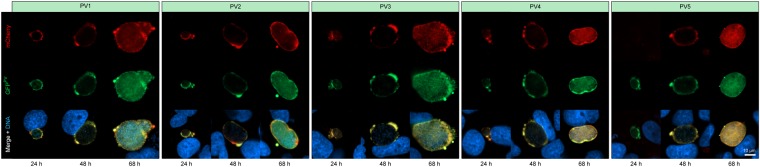
PV protein expression during liver stage development. Transgenic *Plasmodium berghei* parasites expressing fluorescently labelled PV1, 2, 3, 4, or 5 were imaged live throughout *in vitro* liver stage development. Representative parasites were recorded 24, 48, and 68 hours after sporozoite inoculation. Shown are the fluorescent signals of the mCherry-3xMyc-tagged PV proteins (red, top), the marker protein GFP^PV^ (green, middle), as well as a merge of both fluorescent protein signals with Hoechst 33342 nuclear dye (DNA, blue, bottom).

We next wanted to know whether *PV1-5* are also expressed during the liver stage of infection, during which the parasite also resides inside a PV. To that end, we passed the tagged parasite lines through the *Plasmodium* life cycle by feeding highly infected mice to female *Anopheles stephensi* mosquitoes. Inoculation of *in vitro* cultivated hepatoma cells with the transgenic sporozoites revealed that all five proteins are highly expressed in the liver stage PV (Fig. 4). Interestingly, PV5 was not expressed during early pre-erythrocytic growth, but was only present in the PV of more mature parasite stages (Fig. 4).

### *PV5* is essential during asexual blood stage development

**Figure 5:**
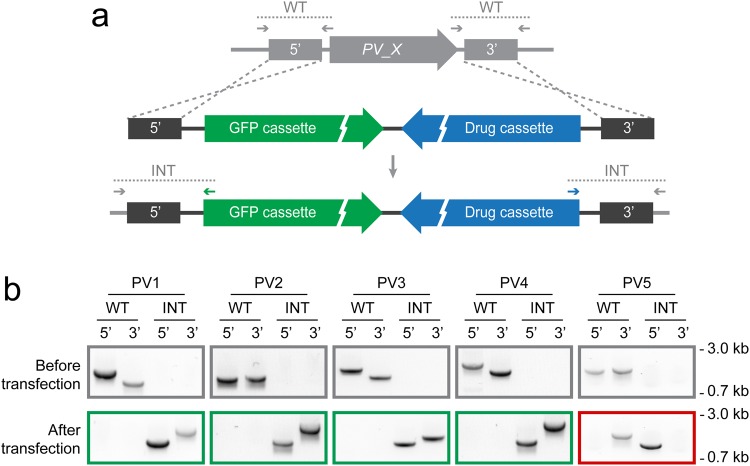
*PV5* is essential during blood infection, whereas *PV1–4* are dispensable. (**a**) Replacement strategy to delete the genes encoding PV proteins of *Plasmodium berghei*. The loci were targeted with replacement plasmids containing the 5′ and 3′ regions flanking the respective open reading frame, a cytoplasmic GFP expression cassette, and the drug-selectable hDHFR-yFcu cassette (drug cassette). For PV visualization, GFP was exchanged for GFP^PV^ (see also Supplementary Fig. S3). Wild-type (WT) and integration-specific primer combinations (INT) are indicated by arrows and expected fragments by dotted lines. (**b**) For each target gene, diagnostic PCRs of the WT locus (top) and of the drug-selected and isolated parasites (bottom) are shown using the primer combinations depicted in a. The green frames denote the successful isolation of loss-of-function mutants. The red frame indicates that three independent transfection experiments did not result in the recovery of a *PV5* deletion strain. For full size images of DNA gels, see Supplementary Fig. S5.

In order to gain insights into the PV proteins’ functions we targeted the respective genes with deletion constructs (Fig. 5a). We were able to select and isolate transgenic *P. berghei* parasites lacking expression of either *PV1*, *2*, *3*, or *4*, as confirmed by diagnostic PCR (Fig. 5b). In contrast, three independent transfection experiments failed to achieve *PV5* deletion. Instead, we obtained parasites that have integrated the targeting construct on the 5′ side but retained the WT 3′ flanking region (Fig. 5b). We have previously observed this phenomenon and reported that this is due to initial single-sided homologous recombination followed by non-homologous endjoining^24^. These rare events leave the gene intact and are favoured during positive selection when essential genes are targeted. Furthermore, the successful endogenous tagging of *PV5* demonstrates that our failure to achieve gene deletion is not due to inaccessibility of the locus. Our findings are further supported by a recent genome-scale mutagenesis study, which revealed that the corresponding *P. falciparum PV5* locus is refractory to transposon insertion^25^. Together, these data strongly support essential functions of *PV5* during asexual blood stage development *in vivo* and *in vitro*.

### PV1-4 do not promote vital PV functions

**Figure 6:**
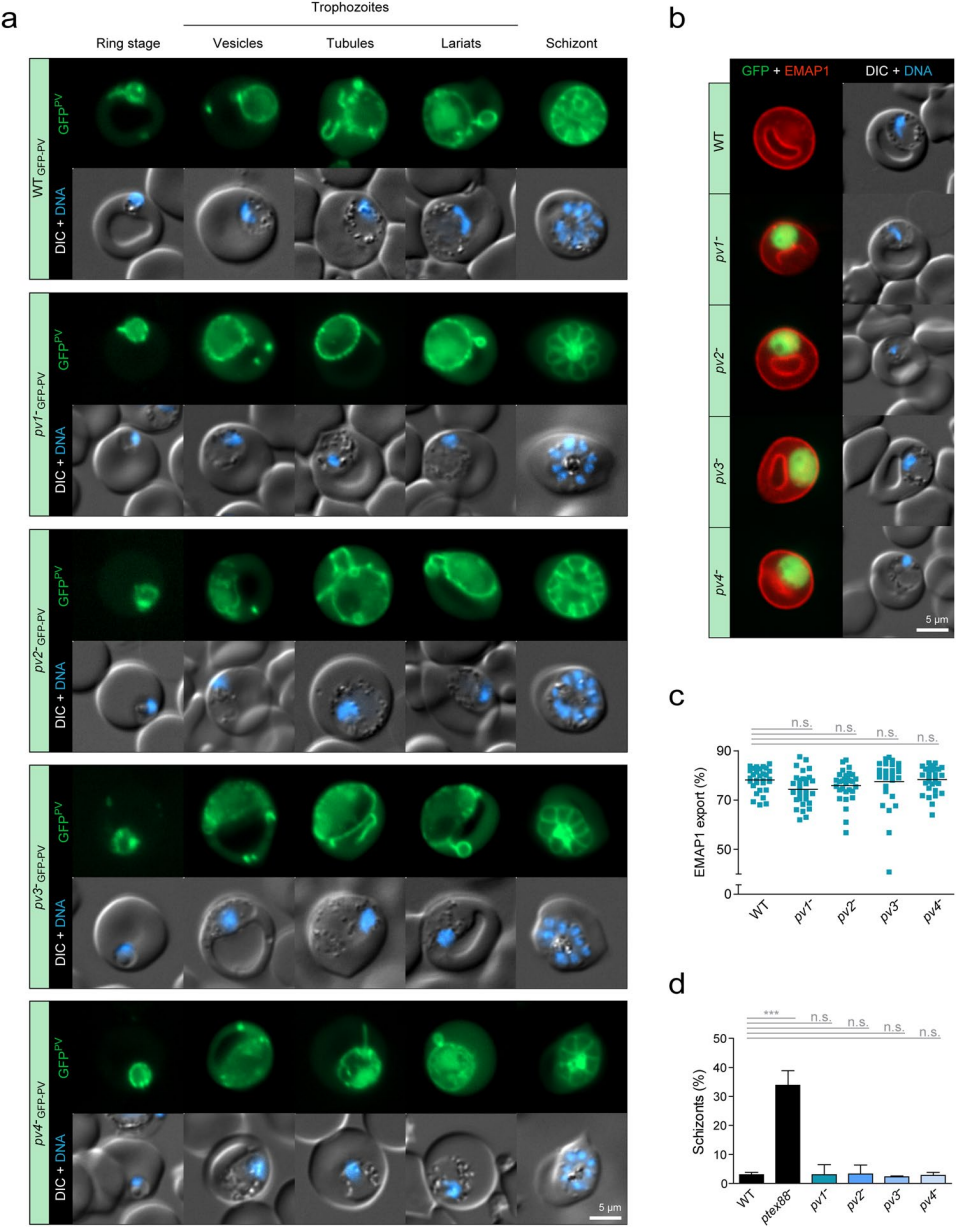
Normal PV morphology and protein export competence in the absence of *PV1*, *2*, *3*, or *4*. (**a**) PV morphology remains unaltered in the absence of *PV1*, *2*, *3*, or *4*. Shown are WT or *PV1*, *2*, *3*, or *4* gene deletion mutants expressing GFP^PV^. Depicted are the fluorescent signal of GFP^PV^ (green, top) and a merge of differential interference contrast images (DIC) with Hoechst 33342 nuclear dye (DNA, blue, bottom). Shown are representative micrographs of asexual blood stages during the ring, trophozoite and schizont stages, as well as selected PV features. (**b**) Export of EMAP1 is not impaired in the absence of *PV1*, *2*, *3*, or *4*. Depicted are representative micrographs of WT or PV knockout parasites expressing the exported mCherry-tagged erythrocyte membrane-associated protein 1 (EMAP1). Shown are a merge of EMAP1 (red) with the cytoplasmic GFP fluorescence of the PV knockout mutants (green, left) and a merge of DIC images with Hoechst 33342 nuclear dye (DNA, blue, right). (**c**) Quantification of EMAP1 export. The extra-parasitic EMAP1-mCherry fluorescence was normalized to the overall EMAP1-mCherry fluorescence of the infected red blood cell. Lines show mean values. n.s., non-significant; One-way ANOVA and Tukey’s multiple comparison test, n = 30. (**d**) Normal sequestration in the absence of *PV1*, *2*, *3*, or *4*. Purified WT, *pv1*^−^, *pv2*^−^, *pv3*^−^, and *pv4*^−^ schizonts were injected intravenously into naïve mice and the number of circulating multi-nucleated parasites was determined 22 hours later. The *ptex88*^−^ line was reported to exhibit reduced sequestration *in vivo* and served as a positive control^26^. Shown are mean values (±SD). ***P < 0.001; n.s., non-significant; One-way ANOVA and Tukey’s multiple comparison test, n = 3.

We then went on to test the involvement of *PV1*-*4* in signature PV functions and first assessed vacuolar morphology. We generated independent gene deletion lines expressing GFP^PV^ (Supplementary Fig. S3) and examined signature PV formations including vesicles, tubules and lariats^19^. All PV features were present despite the absence of *PV1*, *2*, *3*, or *4*, and general PV morphology appeared normal throughout asexual blood stage development (Fig. 6a).

We then asked whether these proteins might promote the export of virulence factors across the PVM and, thus, quantified the export of an exemplary cargo protein. To that end, we crossed the knockout mutants *in vivo* with a transgenic parasite line expressing mCherry-tagged erythrocyte membrane-associated protein 1 (EMAP1) as demonstrated previously^26^. The protein was efficiently exported by all parasite lines as shown by live fluorescence imaging and quantitative microscopy, suggesting general protein export competence in the absence of *PV1*, *2*, *3*, or *4* (Fig. 6b,c). To exclude that other exported proteins may yet be retained in the parasite upon deletion of *PV1*, *2*, *3*, or *4* we used iRBC sequestration as a proxy for the parasite’s general protein export status. Since deficiencies in protein translocation across the PVM are expected to result in reduced iRBC sequestration to peripheral organs, we quantified the circulating schizonts during synchronized *in vivo* infections (Fig. 6d). As expected, only very few WT schizonts were detected in the circulation as most of them had sequestered to the peripheral organs, *e.g*. lungs, brain and adipose tissue^26,27^. In contrast, a transgenic parasite line lacking expression of the protein export regulator PTEX88 showed a marked elevation of circulating schizonts (Fig. 6d)^26^. The parasite lines lacking *PV1–4*, however, showed normal sequestration behaviour in agreement with their complete protein export competence (Fig. 6d).

### Dispensable roles of *PV1-4* during life cycle progression

**Figure 7:**
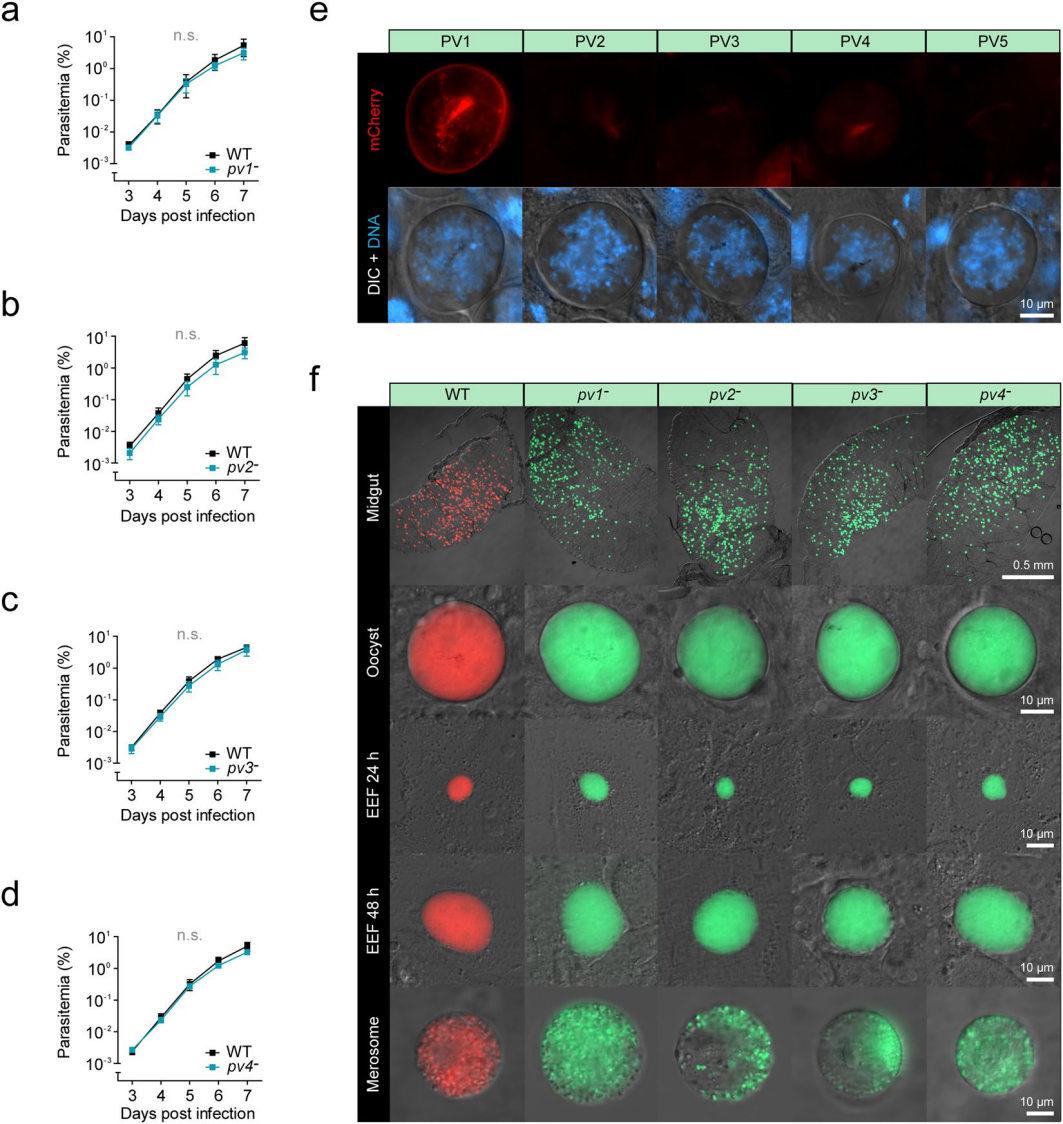
*PV1–4* do not promote parasite fitness during life cycle progression. (**a–d**) Intravital competition assay demonstrates normal blood propagation in the absence of *PV1* (**a**), *PV2* (**b**), *PV3* (**c**), or *PV4* (**d**). 500 mCherry-fluorescent Berred WT and 500 GFP-fluorescent knockout blood stage parasites were co-injected into NMRI mice and peripheral blood was analysed daily by flow cytometry. Mean values (±SD) are shown for each time point. n.s., non-significant; Two-way ANOVA, n = 3. (**e**) PV1 is expressed during the oocyst stage. Shown are transgenic *P. berghei* parasites expressing the endogenous PV proteins fused to mCherry-3xMyc (red, top) as well as a merge of differential interference contrast images (DIC) with Hoechst 33342 nuclear dye (DNA, blue, bottom). Depicted are 10 day old oocysts. (**f**) Normal maturation of PV knockout parasites in the mosquito and in hepatocytes. Representative live fluorescence micrographs of Berred wild-type (WT) or PV knockout parasites during the oocyst and liver stages. Shown is a merge of the parasite’s cytoplasmic fluorescence (WT, mCherry, red; PV knockouts, GFP, green) and differential interference contrast images (DIC). Midgut-associated oocysts were recorded 10 days after the blood meal, liver stages were recorded 24, 48 and 72 hours after sporozoite inoculation.

Next, we asked whether *PV1-4* have an impact on parasite fitness during asexual blood propagation. Therefore, we employed the intravital competition assay to analyse the gene deletion mutants’ expansion in the blood stream^24^. In the absence of *PV1*, *2*, *3*, or *4*, parasites grew indistinguishable from WT during *in vivo* infections, indicating that these genes are entirely dispensable during asexual blood propagation (Fig. 7a–d). We then went on to assess the importance of the vacuolar proteins during parasite life cycle progression. Due to the extracellular nature and the absence of a PV during the oocyst stage, we expected the PV proteins to be switched off throughout mosquito infection. While this was true for PV2–5, PV1 was highly expressed in the periphery of growing oocysts (Fig. 7e). Nonetheless, mutants devoid of *PV1*, *2*, *3*, or *4* all formed high numbers of oocysts displaying a similar morphology as the WT (Fig. 7f). Enumeration of *pv1*^*−*^, *pv2*^*−*^, *pv3*^*−*^, and *pv4*^*−*^ oocysts and salivary gland-associated sporozoites in infected *A. stephensi* mosquitoes revealed similar numbers in comparison to infections with WT parasites (Fig. S4).

We also isolated salivary gland-associated sporozoites for the infection of human hepatoma cells (Huh7) *in vitro*. In the absence of *PV1*, *2*, *3*, or *4*, liver stages matured normally and gave rise to detaching merosomes (Fig. 7f). We then allowed infected mosquitoes to feed on C57BL/6 mice and monitored peripheral blood daily by microscopic examination of Giemsa-stained thin blood films. All animals bitten by WT or knockout-infected mosquitoes became blood-stage positive on day 3 after bite back (n = 3).

Together, our data show that *PV1–4* have dispensable roles for *Plasmodium* life cycle progression, despite their abundant expression and localisation to the PV of liver stages and propagating asexual blood stages.

## Discussion

The plasmodial PV proteome remains a largely unresolved and important frontier in malaria research. While many parasite-encoded proteins of the compartment’s surface, the PVM, have been identified and characterized^28^, the composition of the PV matrix remains less well understood. Until recently biochemical approaches have been employed to identify soluble PV proteins. Differential permeabilization of *Plasmodium falciparum*-infected erythrocytes combined with biotin labelling uncovered the presence of PV1^17^. A more recent study on *Plasmodium yoelii* employed a label-free subcellular fractionation method combined with a subsequent protein distribution analysis and identified several new peripheral parasite proteins^14^. Only recently, the implementation of proximity-dependent biotinylation has led to the identification of additional vacuolar proteins in *P. falciparum*^12^ and *P. berghei*^13^. While all these studies have significantly added to our scarce knowledge of the vacuolar composition, the inherent methodological limitations of such biochemical approaches have led to high misprediction rates, bringing the generation of a PV proteomic map far beyond our reach. It is, thus, desirable to employ complementary approaches to obtain a broader view of the molecular make-up of the PV.

We, for the first time, used an alternative *in silico* strategy, which took advantage of refined prediction algorithms, to identify three previously unrecognised abundant *Plasmodium* proteins in the PV. In order to achieve the highest possible predictive accuracy, we have applied very stringent exclusion criteria, which inevitably led to the omission of many known, and perhaps also undetermined, PV proteins. These include all schizont and gametocyte-specific PV proteins as well as such with erroneous protein export or apicoplast targeting predictions. Furthermore, our approach was exclusively aimed at soluble PV proteins, since transmembrane domains can target secreted proteins to multiple locations in the secretory pathway^29^. Taking proteins into account that were removed from the final selection due to their already reported PV localisation, namely PTEX150, PTEX88, p1/s1 nuclease, and glycerophosphodiester phosphodiesterase^14,30,31^, our positive prediction rate was ~60% and led to the identification of three novel PV proteins.

We point out that the validation of putative PV proteins requires special attention. If proteins are tagged at the carboxy-terminus, ER retention signals become unrecognisable leading to erroneous secretion of the fusion proteins. We present a straightforward way of assessing this possibility and in the process identified a putative protein disulfide isomerase, PBANKA_0820300, which harbours a cryptic ER retention signal. Furthermore, secreted proteins can associate with the PV(M) and/or with the parasite surface. We excluded association of the PV proteins with the parasite plasma membrane by imaging emerging merozoites. In addition, peripheral protein localisation patterns with simultaneous exclusion from PV emergences suggest an association with the parasite surface, as shown for PBANKA_0827000.

We were surprised to find that, despite their high-level expression in blood and liver stages, *PV1–4* can be ablated without any apparent effect on parasite fitness *in vivo*. It remains possible that these proteins perform yet unrecognised functions during certain conditions, *e.g*. during host malnutrition, fever, or long-term infection. Alternatively, many abundant and redundant soluble proteins might provide a favourable crowding effect^32^ for vital functional pathways of the PV, which is not impaired by the loss of one individual factor. The generation of mutant strains lacking several PV proteins could help to unravel cumulating fitness costs upon successive depletion of potential ‘PV filler material’.

PV1 and 2 were previously reported to be part of an exported protein-interacting complex (EPIC)^18^. Interestingly, ablation of *P. falciparum PV1* was reported to result in modest defects in protein export, cytoadhesion, rigidity, and surface remodelling of the iRBC. However, these defects did neither influence *in vitro* growth of *P. falciparum* nor did they translate to a fitness loss during *in vivo* infections with transgenic *PV1*-deficient *P. berghei* parasites^18^. We corroborate the authors’ findings that *PV1* and *2* are dispensable for rapid blood-stage development *in vivo*. We provide evidence that *P. berghei* parasites lacking *PV1* or *2* are protein export competent and that they sequester normally to peripheral tissues. It could be argued that *P. falciparum* exports considerably more proteins to the iRBC than *P. berghei*^33–35^ and, thus, consequences of depletion are expected to be more severe for the human pathogen. Furthermore, protein export in *PV1*-deficient *P. falciparum* parasites had previously only been quantified using species-specific cargo, namely *P. falciparum* erythrocyte membrane proteins 1 and 3 (*Pf*EMP1/3) and knob-associated histidine-rich protein (KAHRP), which are absent in *P. berghei*^18^. We wish to note that we also observed PV1, but none of the other PV proteins, in the periphery of developing oocysts in the mosquito vector, the only extracellular replication phase in the *Plasmodium* life cycle, which is considered to be devoid of a PV. It remains unclear why PV1 is secreted during this stage, but its ablation did not cause any defects during sporogonic development.

The newly identified PV3 and 4 are also dispensable throughout the entire parasite life cycle and the elucidation of their molecular functions remains challenging in the absence of an observable effect upon gene deletion. Protein interaction studies could help to associate these proteins with functional pathways in the PV. *PV3* (PBANKA_1350500) encodes a hypothetical protein of unknown function and contains a high proportion of charged amino acid residues (35%). *PV4* (PBANKA_1349200) harbours in its carboxy-terminal portion the key signatures of a merozoite surface protein 7-like protein. Its localisation throughout blood infection, however, suggests functions predominantly in the PV, as previously shown for another proposed merozoite surface protein, MSP8^36,37^. Interestingly, inactivation of MSP8 had no effect on blood stage growth or protein export of *P. berghei* either^37^. Surprisingly, we observed a significant portion of PV4 in the erythrocyte cytoplasm during the ring and young trophozoite stages, despite the lack of any discernable targeting information. This exported fraction was lost during more mature parasite stages, possibly signifying different functions during early and late intraerythrocytic development. While evidence for this is very circumstantial, it would be an attractive scenario if redundant proteins of the merozoite surface have also served as a pool for the evolution of novel vacuolar and exported proteins during *Plasmodium* anagenesis.

We have also identified one protein (PBANKA_0826700) that appears to be essential during asexual blood stage development, which we termed PV5. The peculiar integration of the gene deletion construct strongly suggests indispensable functions for this gene. In addition, the survival of the tagged parasite line argues for the accessibility of the locus and validates our assessment of gene essentiality. The orthologous *P. falciparum PV5* (PF3D7_0925900) encodes a hypothetical protein of 217 amino acids and was previously found to be resistant to mutagenesis in a large-scale experimental genetics screen using cultivated blood stages^25^. Accordingly, the functional investigation of this novel and critical PV protein should be a priority for future investigations.

In conclusion, we present an *in silico* down-scaling approach to identify one essential and two dispensable PV proteins and prove how refined prediction algorithms can complement biochemical approaches to map protein subcellular location in apicomplexan parasites, as exemplified here for *Plasmodium* PV proteins.

## Methods

### Experimental animals

This study was carried out in strict accordance with the German ‘Tierschutzgesetz in der Fassung vom 22. Juli 2009’ and the Directive 2010/63/EU of the European Parliament and Council ‘On the protection of animals used for scientific purposes’. The protocol was approved by the ethics committee of the Berlin state authority (‘Landesamt für Gesundheit und Soziales Berlin’, permit number G0294/15). C57BL/6 mice were used for sporozoite infections. All other parasite infections were conducted with NMRI mice.

### Generation and validation of recombinant parasite lines

We used advanced experimental genetic techniques to generate and isolate all recombinant parasite lines^38–40^. Further details on vector construction and genotyping strategies, including primer sequences and restriction endonuclease recognition sites used for molecular cloning, are provided in Figs 2a and 5a, Supplementary Fig. S1, and Supplementary Table S1. For the generation of gene deletion constructs, 3′ fragments were amplified from genomic DNA (ranging in size from 837 to 1,020 bp) and cloned into the pBAT-G6^24^ or the pGFP-PV plasmid^19^ using the XhoI and KpnI restriction sites. Subsequently, 5′ fragments (ranging in size from 808 to 1,003 bp) were amplified and cloned into the intermediate vectors using SacII in combination with EcoRI or PvuII.

For endogenous tagging of the PV candidates, a GFP^PV^ co-localisation plasmid was constructed by fusing the bacterial backbone and adjacent mCherry-3xMyc tag sequence of the pBAT vector^40^ with the *Plasmodium*-specific fraction of the pGFP-PV plasmid^19^ using the KpnI and PvuII restriction sites. The sequences directly upstream of the stop codons were amplified from genomic DNA (ranging in size from 758 to 1,628 bp) and inserted into the GFP^PV^ co-localisation plasmid using HpaI in combination with EcoRI or SacII. For double homologous recombination, 3′ fragments were amplified and cloned into the intermediate vectors as described for the gene deletion constructs.

Prior to transfection into wild-type ANKA parasites, knockout vectors were digested with XmnI and SalI. Linearization of the tagging vectors was performed with ApaLI and AhdI (double homologous recombination) or with internal endonuclease restriction sites (single homologous recombination). To validate correct integration of the transfection vectors and absence of contaminating WT parasites in the isogenic recombinant parasite lines, primer combinations were used as indicated in Figs 2a and 5a, Supplementary Fig. S1, and Supplementary Table S1.

In order to test protein export competence, gene deletion mutants were crossed in the mosquito with the *emap1-mCherry* line, as described previously^26^.

### *In vivo* infections

Asexual blood stage development was assessed using the robust intravital competition assay^24^. In short, 500 knockout blood stage parasites were co-injected intravenously with 500 Berred WT blood stage parasites into NMRI mice and parasitemias were measured by flow cytometry. Sequestration was analysed by injecting purified schizonts intravenously into NMRI mice and quantifying the circulating parasite stages 22 hours later^26^.

In order to test transmission efficiency, NMRI mice were injected intravenously with 10^7^ blood stage parasites and fed to female *A. stephensi* mosquitoes three days later. 21 days after the infectious blood meal, C57BL/6 mice were subjected to bites from >100 infected mosquitoes and blood stage patency was assessed by microscopic analysis of Giemsa-stained thin blood films.

### Live cell imaging

All fluorescence imaging was performed with a Zeiss AxioObserver Z1 epifluorescence microscope equipped with a Zeiss AxioCam MRm camera (Zeiss, Oberkochen, Germany). Tagging and knockout mutants were passed through the parasite life cycle and recorded at different time points. Asexual blood stages were imaged from peripheral blood or *ex vivo* blood cultures. Oocysts were recorded on days 10, and sporozoites on day 21 after the blood meal. Huh7 cells were seeded onto µ-slide 8 well glass bottom slides (Ibidi, Martinsried, Germany), infected with 10,000 sporozoites at subconfluence, and imaged 24, 48, and 72 hours after infection. Nuclei were visualized with Hoechst 33342 nuclear dye (1:1,000).

Export of EMAP1 was assessed by quantifying the raw integrated density (RID) of EMAP1-mCherry associated with the parasite and with the entire infected erythrocyte. Export efficiency was calculated as follows:

$${\rm{y}}=(({{\rm{RID}}}_{{\rm{infected}}{\rm{cell}}}\,-\,{{\rm{RID}}}_{{\rm{parasite}}})/{{\rm{RID}}}_{{\rm{infected}}{\rm{cell}}})\,\ast \,100.$$

## Material and Data Availability

The materials and datasets generated during the current study are available from the corresponding author on reasonable request.

## Electronic supplementary material

Supplementary information
