# Supplementary material for: An in silico down-scaling approach uncovers novel constituents of the Plasmodium-containing vacuole
Source: Sci Rep. 2018 Sep 19;8:14055. doi: 10.1038/s41598-018-32471-6 (PMC6145888; doi:10.1038/s41598-018-32471-6)
Supplement: Supplementary file 1 — Supplementary information [file 41598_2018_32471_MOESM1_ESM.pdf]

SUPPLEMENTARY ONLINE MATERIAL

An *in silico* down-scaling approach uncovers novel constituents of the *Plasmodium*-containing vacuole

Joachim Michael Matz & Kai Matuschewski

Contents:

Supplementary Figure S1: Several candidates show no PV localisation.

Supplementary Figure S2: Expression profiling and live protein localization of PV1-5 throughout asexual blood stage development.

Supplementary Figure S3: Validation of transgenic GFP^{PV}-expressing parasites lacking *PV1*, *2*, *3*, or *4*.

Supplementary Figure S4: Quantification of oocyst and sporozoite numbers indicate no defects during mosquito stage development in the absence of *PV1-4.*

Supplementary Figure S5: Full gel images.

Supplementary Table S1: Primers for the generation and analysis of transgenic parasites.

Supplementary references

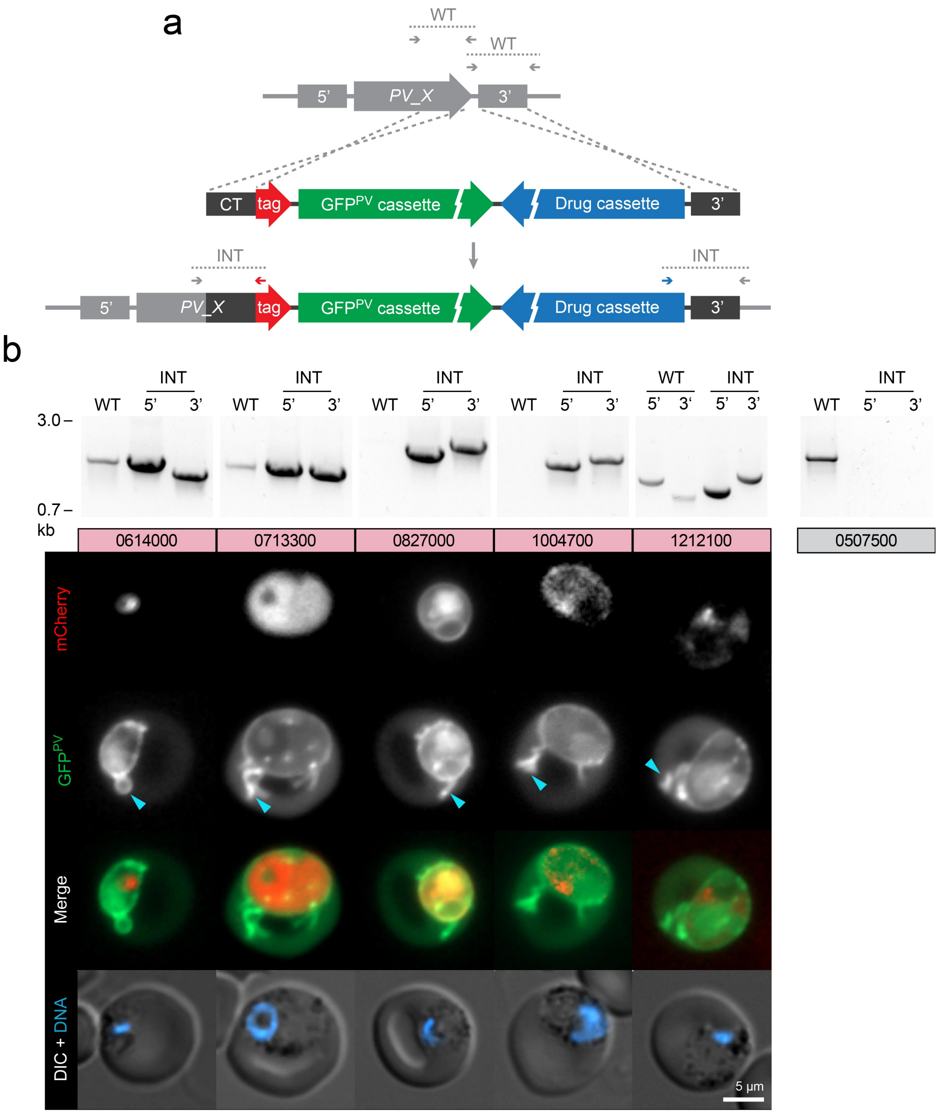

Supplementary Figure S1. Several candidates show no PV localisation. (**a**) Strategy for the generation of transgenic parasite lines expressing endogenous PV proteins (*PV_X*) fused to mCherry-3xMyc (tag) by double homologous integration. In addition, recombinant parasites contain the drug-selectable hDHFR-yFcu cassette (drug cassette) and the GFP^{PV} cassette. Wild-type (WT) and integration-specific (INT) primer combinations (Table S1) are indicated by arrows and expected fragments by dotted lines. Note, that only PV1 and PBANKA_1212100 were tagged using this strategy. All other candidates were tagged by employing single homologous recombination (see Fig. 2a). (**b**) Live fluorescence microscopy of candidate proteins showing no PV localisation. Numbers indicate gene accession codes (without the 'PBANKA_' prefix). Shown are transgenic *P. berghei* blood stage parasites expressing the endogenous candidate genes fused to mCherry-3xMyc (red, top) and GFP^{PV} (green, lower top) as well as a merge of both fluorescent protein signals (upper bottom) and a merge of differential interference contrast images (DIC) with Hoechst 33342 nuclear dye (DNA, blue, bottom). ►, PV protrusions. Diagnostic PCRs as described in a and b are shown above. Note that repeated attempts to tag PBANKA_0507500 were unsuccessful, suggesting vital functions of the carboxy-terminus during asexual blood stage development. For full size images of DNA gels, see Supplementary Fig. S5.

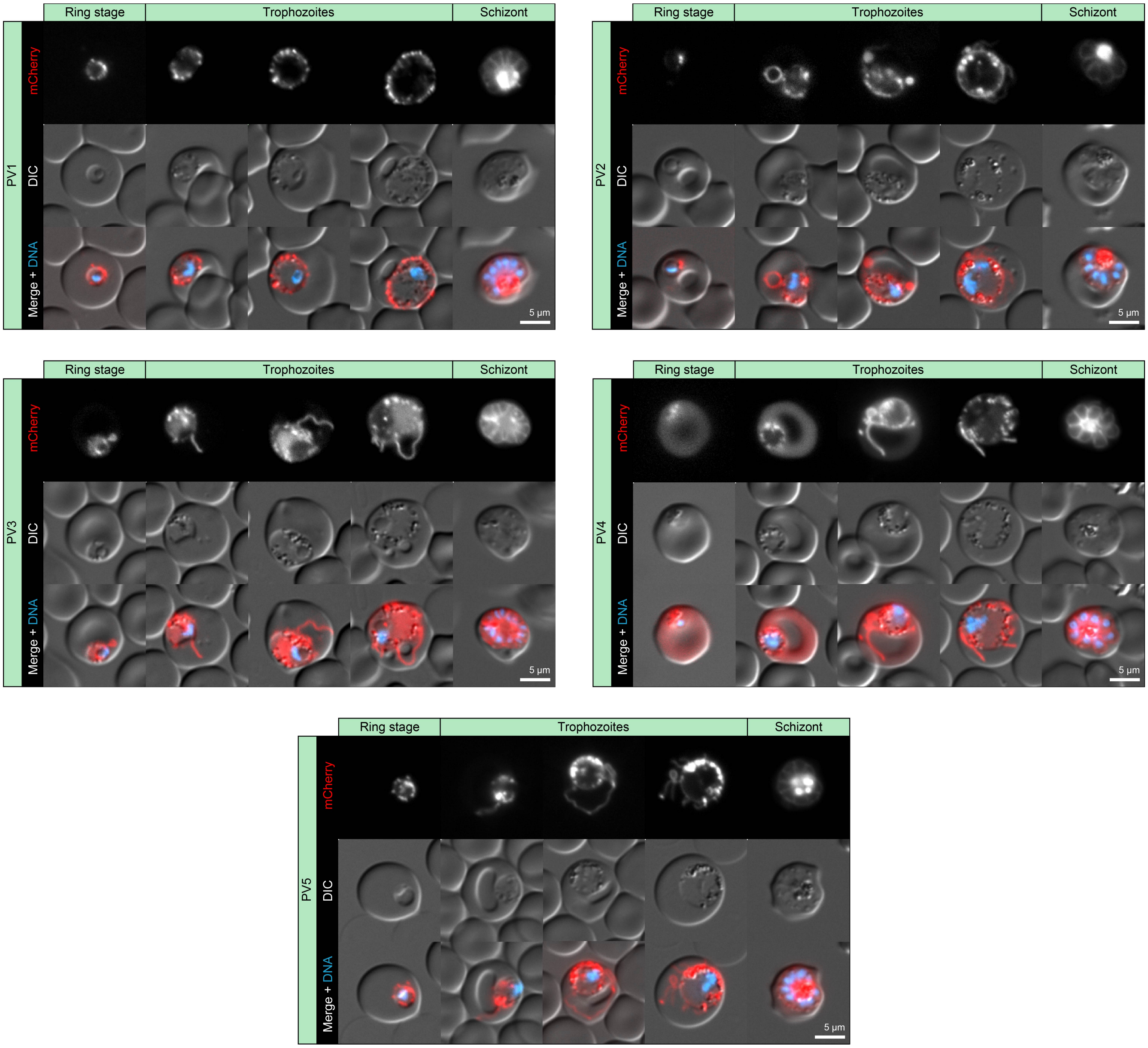

Supplementary Figure S2. Expression profiling and live protein localization of PV1-5 throughout asexual blood stage development. Shown are transgenic *P. berghei* parasites expressing the endogenous *PV1-5* genes fused to mCherry-3xMyc at various stages of the asexual life cycle. In addition to the fluorescent protein (red, top), differential interference contrast images are shown (DIC, middle), as well as a merge of mCherry and DIC with Hoechst 33342 nuclear dye (DNA, blue, bottom).

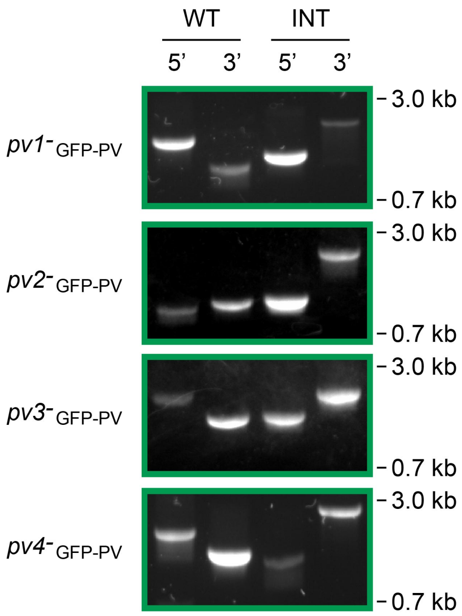

Supplementary Figure S3. Validation of transgenic GFP^{PV}-expressing parasites lacking *PV1*, *2*, *3*, or *4*. For each target gene diagnostic PCRs of the drug-selected parasites are shown using the primer combinations depicted in Figure 5a. Note that the cassette driving high-level expression of cytoplasmic GFP (Fig. 5a) was exchanged for the GFP^{PV} cassette. Green frames denote the successful generation of loss-of-function mutants. For full size images of DNA gels, see Supplementary Fig. S5.

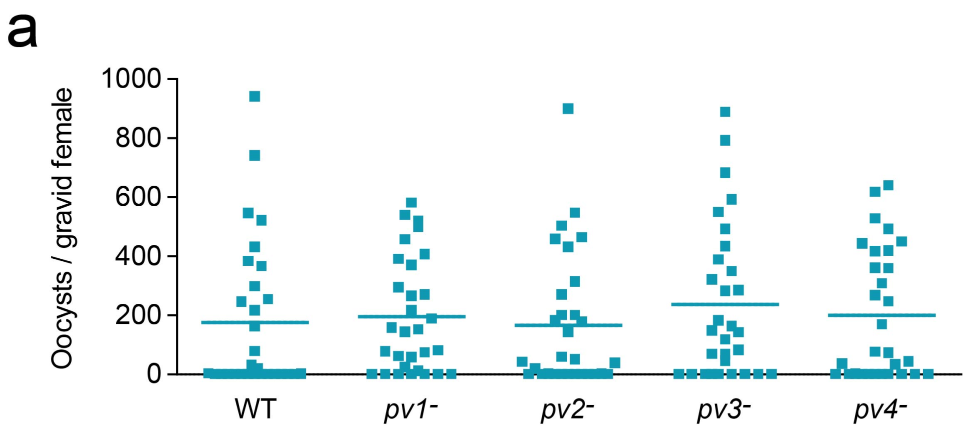

b

	Sporozoites / gravid female
WT	15,256
pv1⁻	8,125
pv2⁻	13,750
pv3⁻	16,375
pv4⁻	8,925

Supplementary Figure S4. Quantification of oocyst and sporozoite numbers indicates no defects during mosquito stage development in the absence of *PV1-4*. (**a**) Midguts of gravid *Anopheles stephensi* females were extracted on day 11 and 12 after the infectious blood meal and oocysts were quantified by live fluorescence microscopy. Lines show mean values. n=30 oocysts. (**b**) Salivary glands were extracted on day 17 after the infectious blood meal and sporozoites were quantified microscopically using a haemocytometer. No statistical analyses were performed, because all collected data are derived from a single feeding experiment.

Supplementary Figure S5

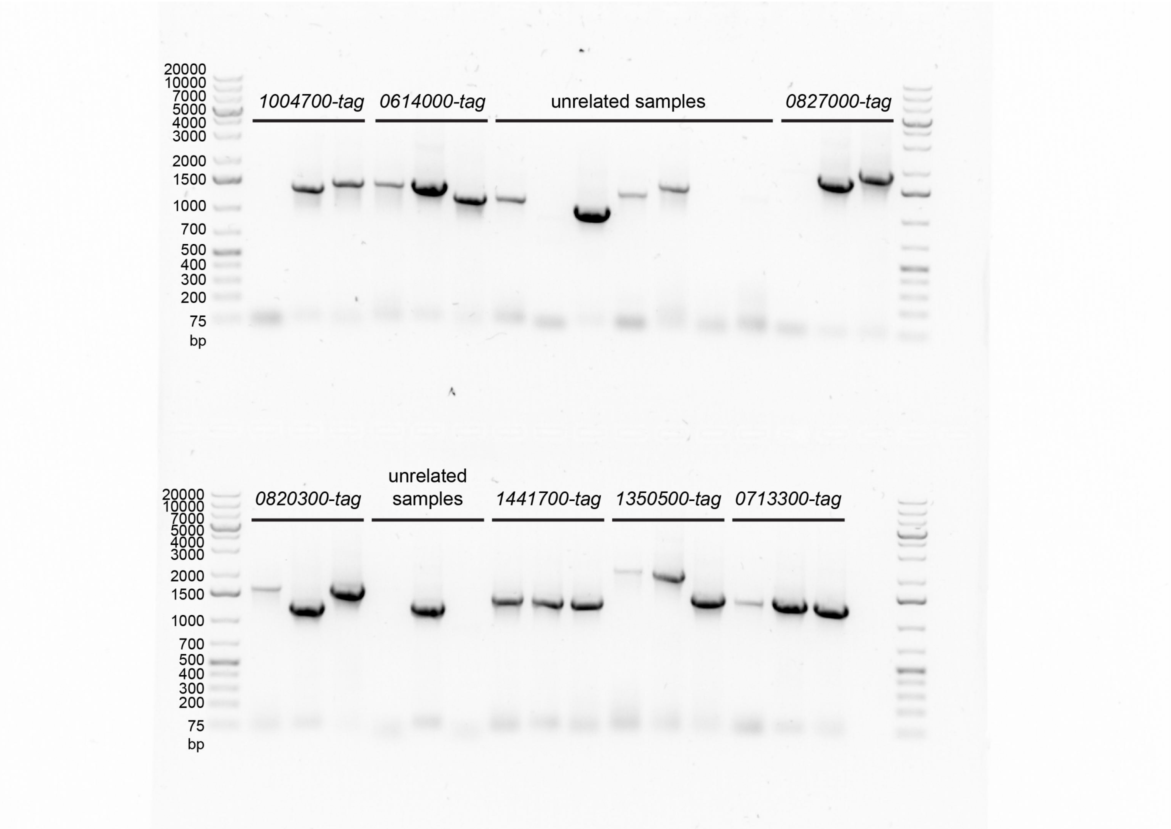

see Figs. 2 and S1

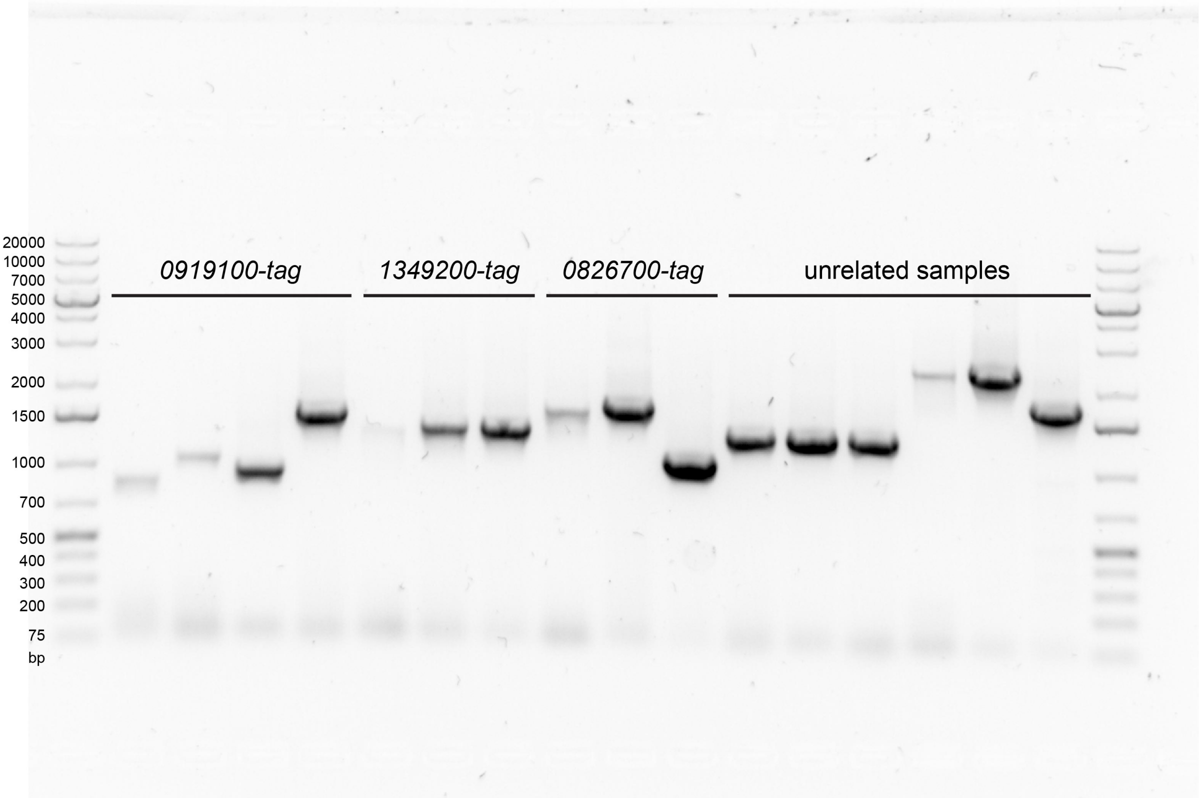

see Figs. 2 and S1

Supplementary Figure S5 (continued)

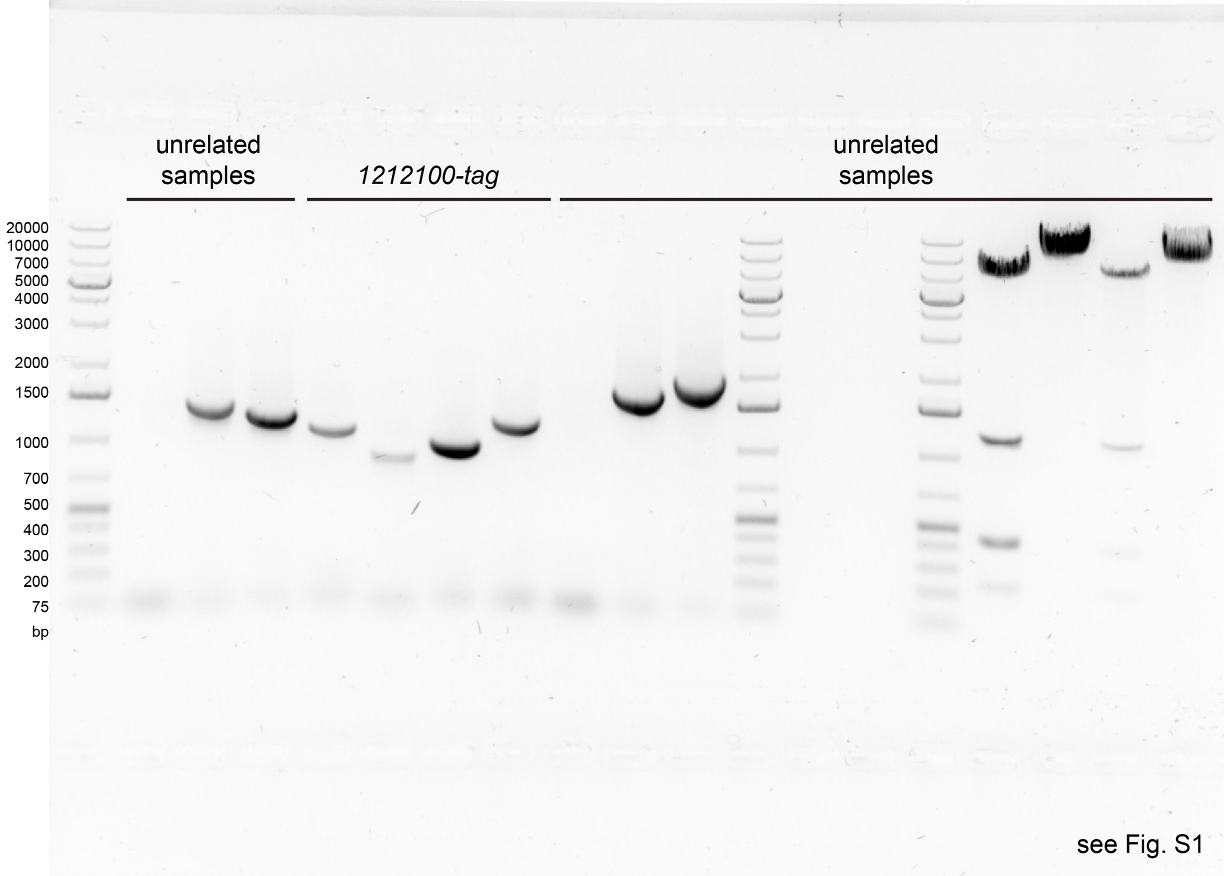

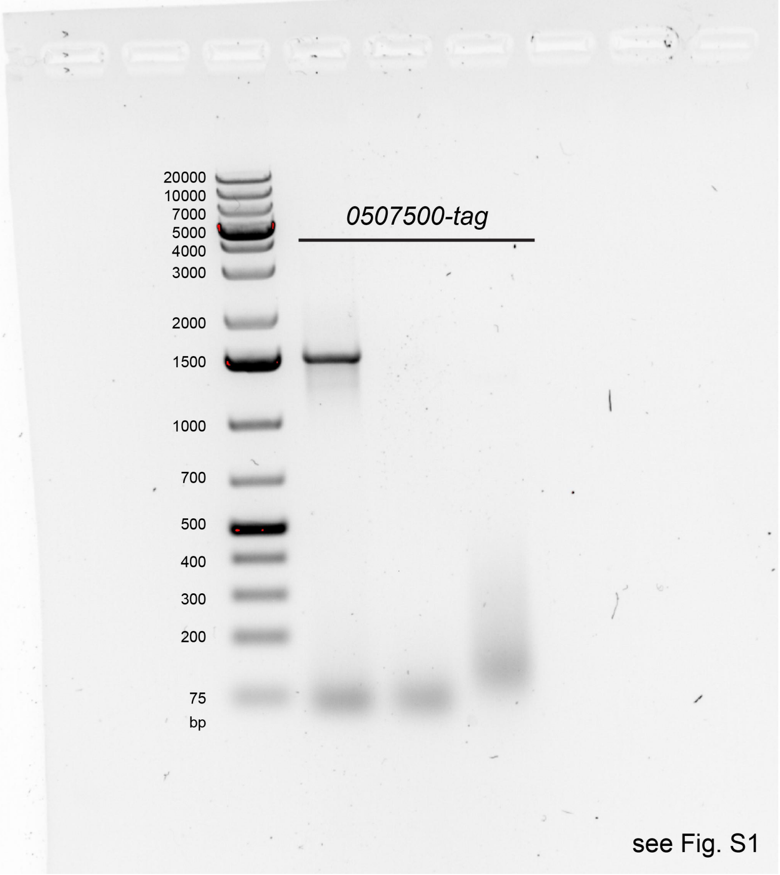

Supplementary Figure S5 (continued)

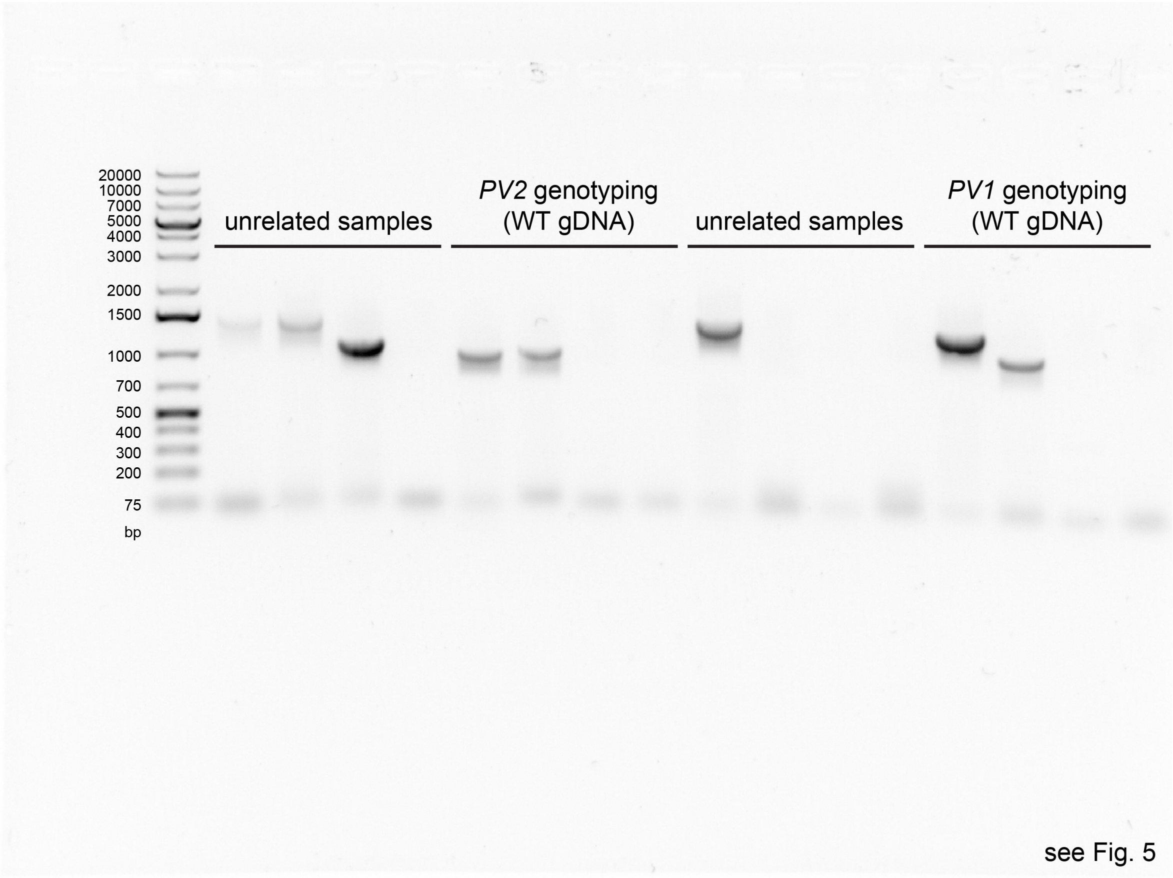

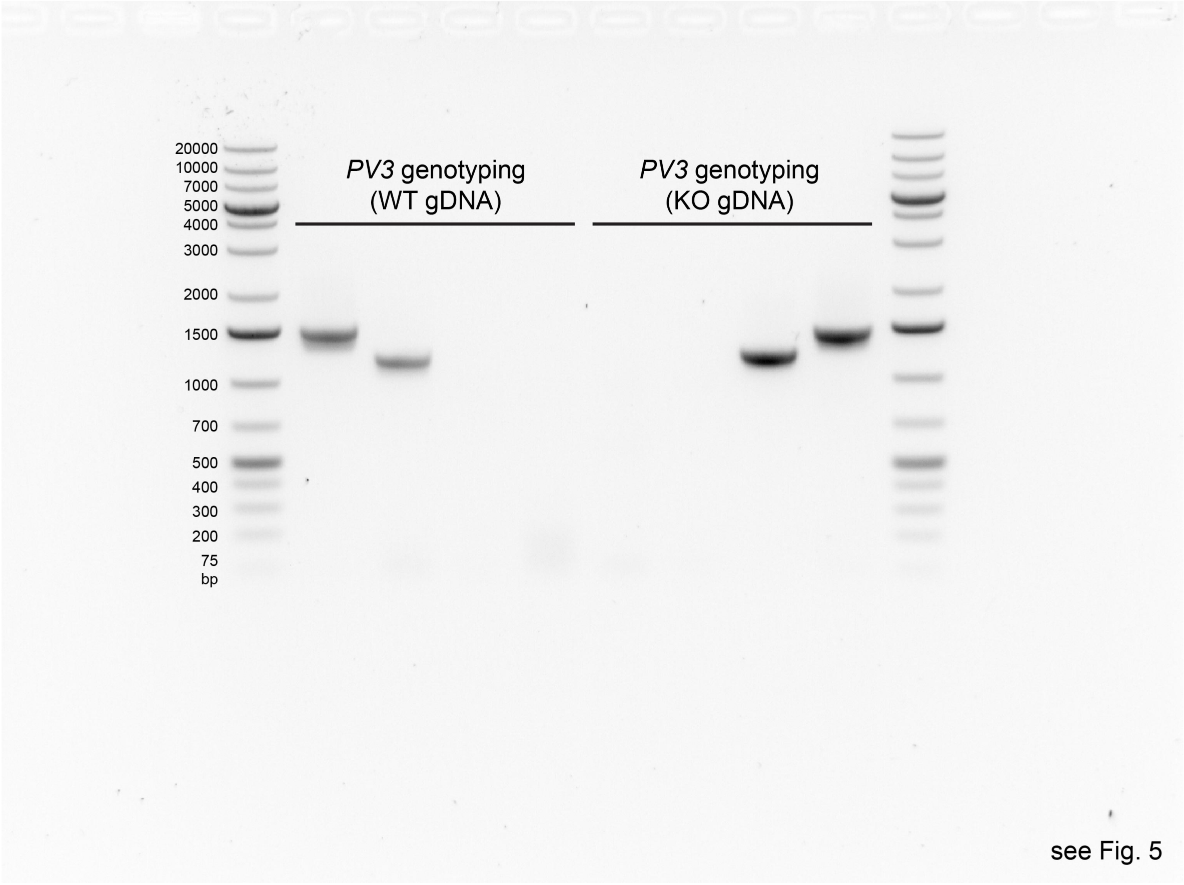

Supplementary Figure S5 (continued)

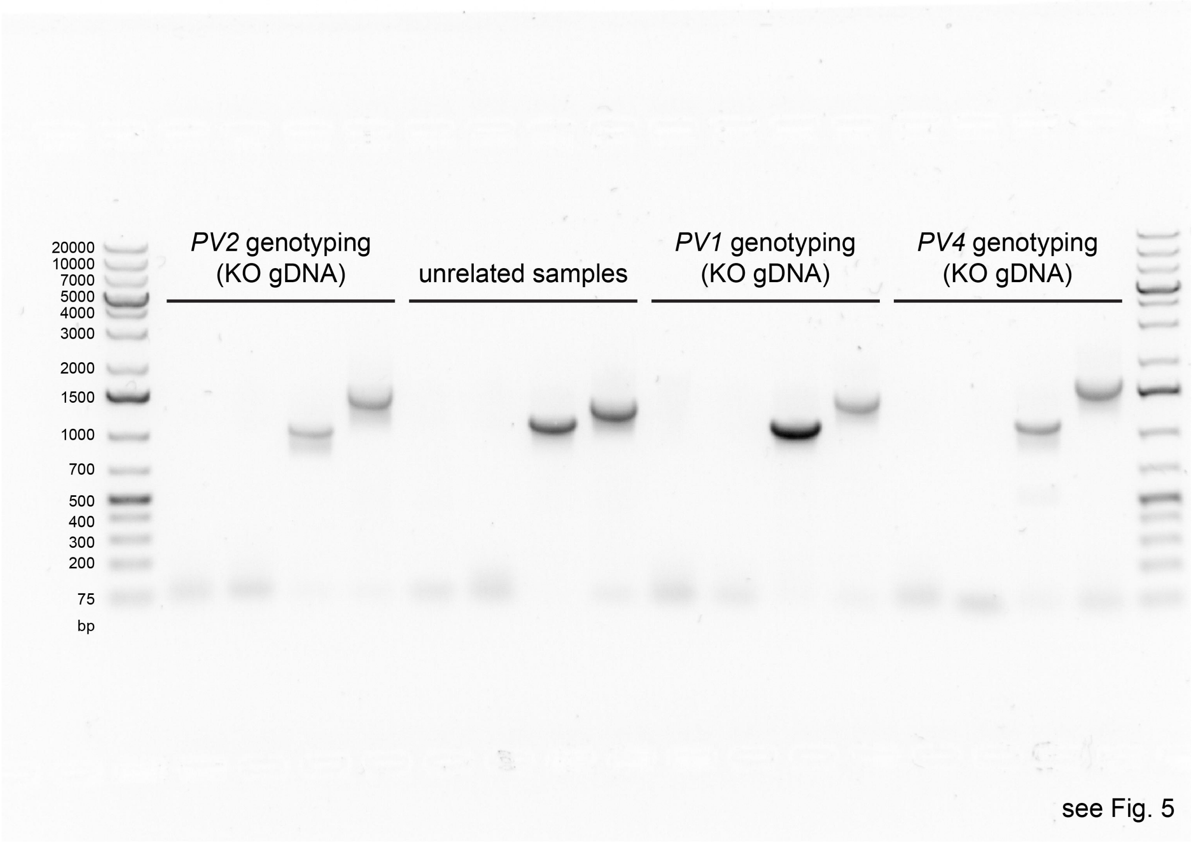

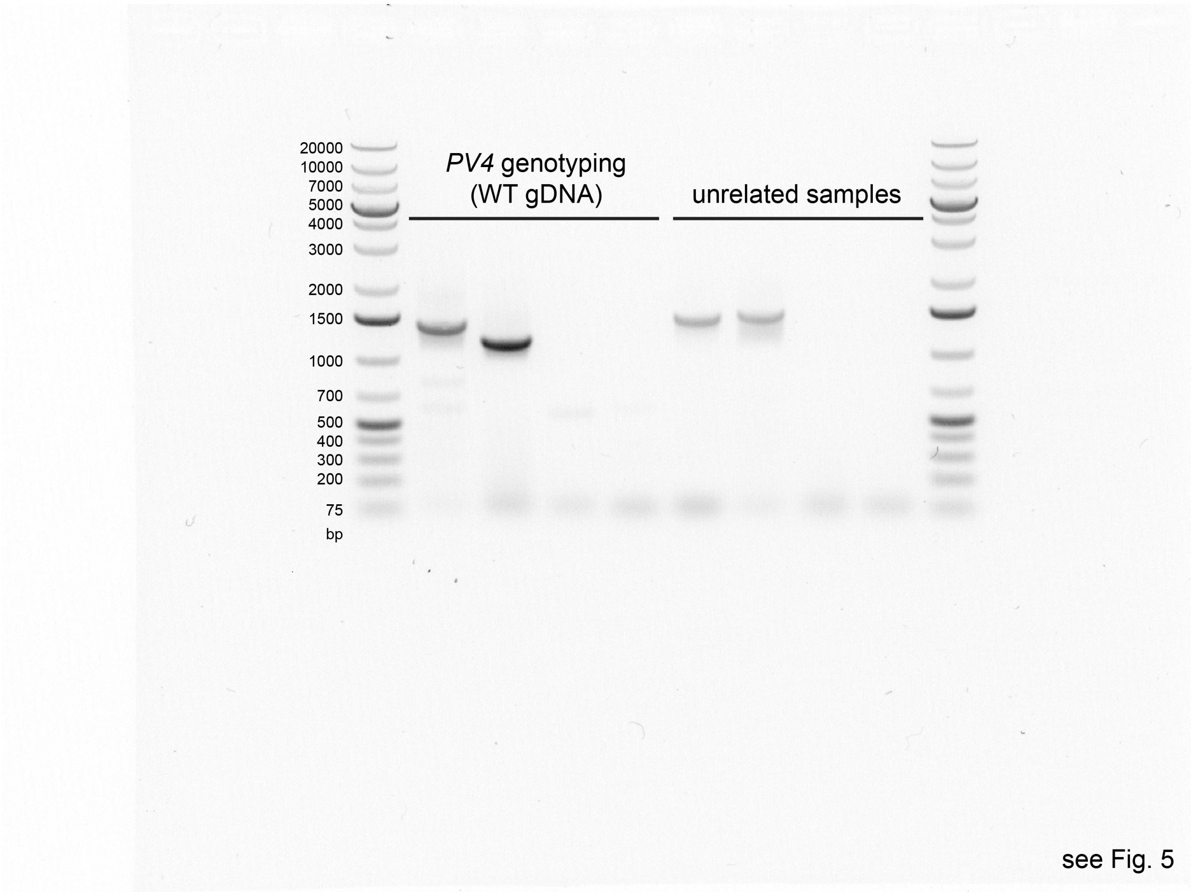

Supplementary Figure S5 (continued)

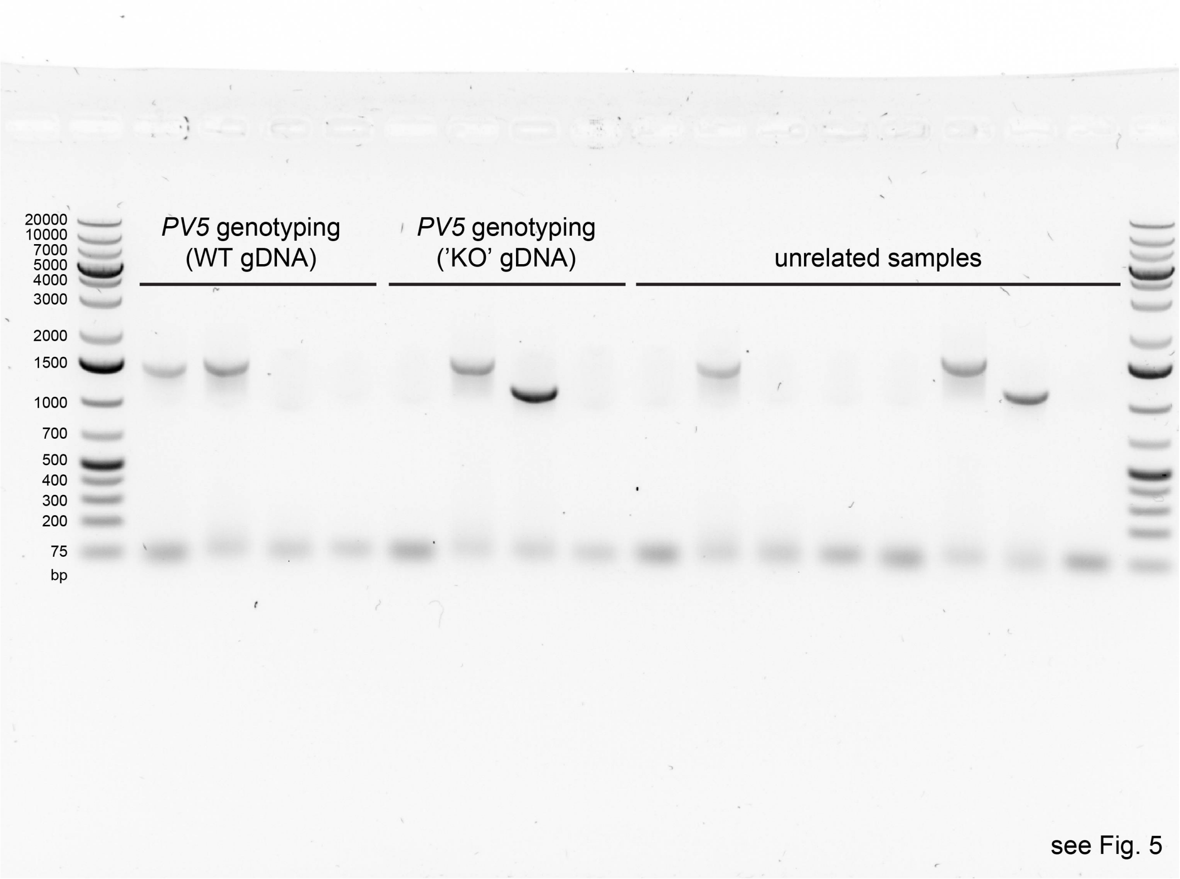

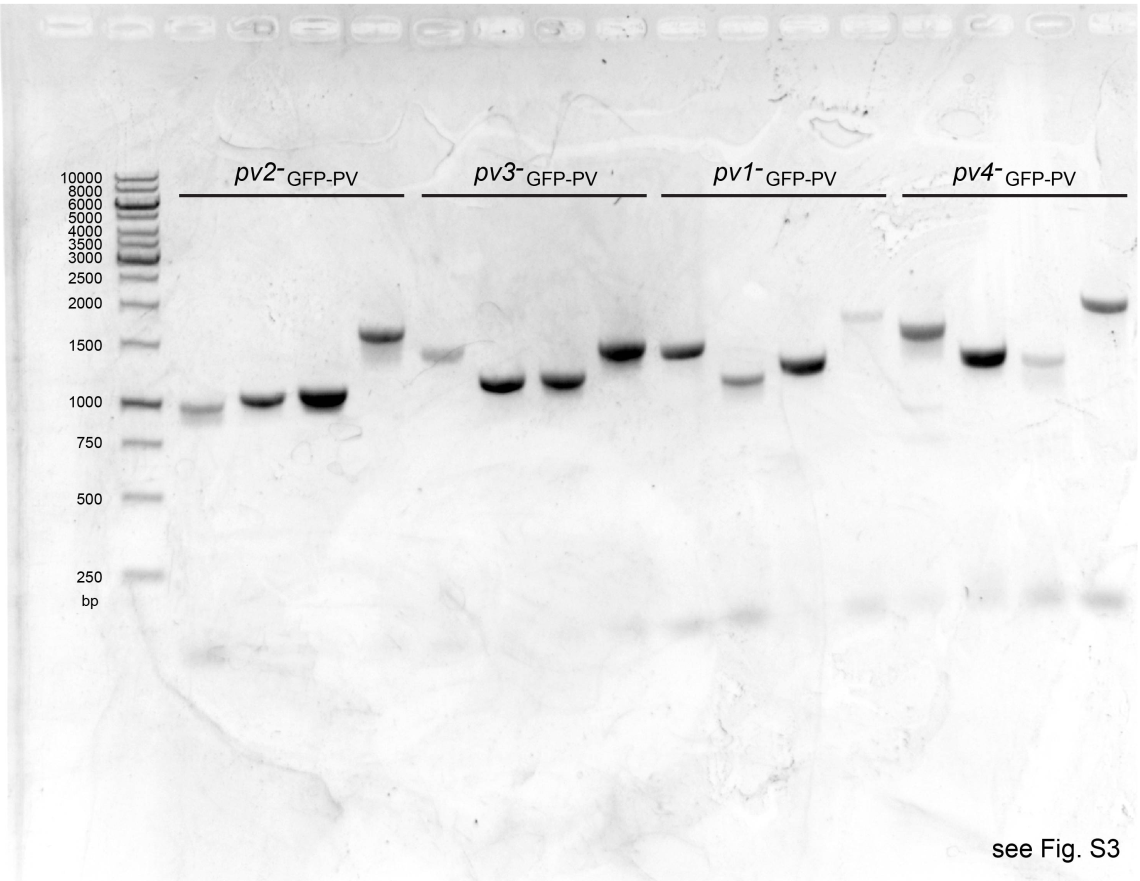

Supplementary Figure S5. Full gel images. Depicted are the full-size agarose gels used for electrophoresis of diagnostic PCRs. Cropped parts of these gels are depicted in Figures 2, 5, S1, and S3.

Supplementary Table S1. Primers for the generation and analysis of transgenic parasites

Primer name	Primer sequence (restriction sites underlined)	Size WT[a]	Size INT[b]	Use[c]	Reference
PBANKA_0507500-CT-F-SacII	TTTATTCCGCGGATATTTTTTTGGAAACATGGAGGTATG	1071 bp		TV (tag)	This study
PBANKA_0507500-CT-R-HpaI	AAAAAAGTTAACTTTTTCTGATATTTGTGTAGTTTCTACAAAATAG			TV (tag)	This study
PBANKA_0614000-CT-F-SacII	AAAAAACCGCGGAATAAAAGCAATGAAAGGGAAATCG	1076 bp		TV (tag)	This study
PBANKA_0614000-CT-R-HpaI	TTTTTAGTTAACAGAAAATATGTCATCATAATTTGTATTGTC			TV (tag)	This study
PBANKA_0713300-CT-F-SacII	AAATAACCGCGGAAGCAACTGCTCCCTTTAACC	1167 bp		TV (tag)	This study
PBANKA_0713300-CT-R-HpaI	AATTATGTTAACTCCGGGGGGCCATTTCATTTG			TV (tag)	This study
PBANKA_0820300-CT-F-SacII	TTTTTTCCGCGGGGGCCCGGTTCTACAATACC	1140 bp		TV (tag)	This study
PBANKA_0820300-CT-R-HpaI	TTAACTGTTAACTAAATCAGAATTTTCTTCTCCTTCTAATATTTC			TV (tag)	This study
PBANKA_0826700-CT-F-SacII	AAATAACCGCGGCAAACAATAATAAAGATTCCCCTGC	973 bp		TV (tag)	This study
PBANKA_0826700-CT-R-HpaI	TTTATAGTTAACATCACAGAAAGTTTGATCAGTTTGG			TV (tag)	This study
PBANKA_0827000-CT-F-SacII	AAATAACCGCGGCAATATAGATAAAAACCACATGCTGC	1628 bp		TV (tag)	This study
PBANKA_0827000-CT-R-HpaI	AACATAGTTAACATCTACTTCAAAATAAAAATCAGTTTTTTTTTTTG			TV (tag)	This study
PBANKA_0919100-3'-F-XhoI	TTTATTCTCGAGTTTGTGTATTTTCGCGTCTGTAC	971 bp		TV (tag)	This study
PBANKA_0919100-3'-R-KpnI	TTTATTGGTACCTGCAGTTGGTTTTTCATATTAACCC			TV (tag)	This study
PBANKA_0919100-CT-F-SacII	ATTTTTCCGCGGCATTTTCAAGATATAGGAGTAGGC	786 bp		TV (tag)	This study
PBANKA_0919100-CT-R-HpaI	TTTTTCGTTAACATTATCTAAATTGGTTTTATCTTGATCAATG			TV (tag)	This study
PBANKA_1004700-CT-F-SacII	TTATTCCGCGGGTTTACGTTTTCCTCCTTTCAAAAGG	1139 bp		TV (tag)	This study
PBANKA_1004700-CT-R-HpaI	TTTTAAGTTAACAGAGTCATTTCTATGAGTATAATAAATAGG			TV (tag)	This study
PBANKA_1212100-3'-F-XhoI	ACTAATCTCGAGTAAAACCAAATGAATAAATATTATACATACATC	739 bp		TV (tag)	This study
PBANKA_1212100-3'-R-KpnI	TTTATTGGTACCGTCTCTCTATCTTTTCCCTTTCC			TV (tag)	This study
PBANKA_1212100-CT-F-SacII	AAATAACCGCGGTAATAGAAGAAAAAAATCTCAAAATCCCC	758 bp		TV (tag)	This study
PBANKA_1212100-CT-R-HpaI	ATACATGTTAACTATTCTTTTCATAAAAATCCTCTTCATAAATAAAAG			TV (tag)	This study
PBANKA_1349200-CT-F-SacII	AAATAACCGCGGCATTAATTATGAAAAGAGGCTCGC	1309 bp		TV (tag)	This study
PBANKA_1349200-CT-R-HpaI	AAACAAGTTAACCATAGAATTAAGTAAGGACAAAACATTTTC			TV (tag)	This study
PBANKA_1350500-CT-F-SacII	ATAAATCCGCGGTGATTATAAATATACCTCATTAAACAGTAGTG	1219 bp		TV (tag)	This study
PBANKA_1350500-CT-R-HpaI	TTATTCGTTAACTTTTTTTTTTTCATTATCTTCATCATCATC			TV (tag)	This study
PBANKA_1441700-CT-F-SacII	AATAATCCGCGGCGATAACCAAGCTGTTGAAGTTTC	1144 bp		TV (tag)	This study
PBANKA_1441700-CT-R-HpaI	AAAATAGTTAACATTGCCTATAATTATACGTTCTATTTGTTTTTC			TV (tag)	This study
PBANKA_0507500-CT-F	ATTTGCTTTAAACTGCTATGCC	1572 bp	1489 bp	GT (tag)	This study
PBANKA_0507500-CT-R	CAATAACAACAAGAAATACTGCACC		1336 bp	GT (tag)	This study
PBANKA_0614000-CT-F	AGAAGACAATCAATTTCAAGAATCC	1493 bp	1488 bp	GT (tag)	This study
PBANKA_0614000-CT-R	TGTCAAAGCGTTTATACATATGC		1261 bp	GT (tag)	This study
PBANKA_0713300-CT-F	CCTATCTAAGTAAGTAACTGAATTGG	1409 bp	1424 bp	GT (tag)	This study
PBANKA_0713300-CT-R	GTCTACGCTTTTTAGCCTCC		1334 bp	GT (tag)	This study
PBANKA_0820300-CT-F	TAAAATACCAAATCTAGCAATCGG	1672 bp	1299 bp	GT (tag)	This study
PBANKA_0820300-CT-R	AGTGAATAAAATAAACAACGCGC		1695 bp	GT (tag)	This study
PBANKA_0826700-CT-F	CGAATTAAAAAGGGAGTAAAGAAAAG	1655 bp	1736 bp	GT (tag)	This study
PBANKA_0826700-CT-R	ATATACATCTTTCACAATTCAACGG		1100 bp	GT (tag)	This study
PBANKA_0827000-CT-F	AATCTGGGTTTATGCATAGAGC	2004 bp	1807 bp	GT (tag)	This study
PBANKA_0827000-CT-R	CCCATAATTTGGTTGTCATTTCG		2007 bp	GT (tag)	This study
PBANKA_0919100-3'-F	GCCTACCTCATGAAAATATGAACC	1080 bp		GT (tag)	This study
PBANKA_0919100-3'-R	GTCTTCAGCATTTAGATGTTGC		1600 bp	GT (tag)	This study
PBANKA_0919100-CT-F	AAGGGACTACAAATTATTGTTAATGC	885 bp	951 bp	GT (tag)	This study
PBANKA_0919100-CT-R	TGATACTAATGTACAGACGCG			GT (tag)	This study
PBANKA_1004700-CT-F	TTAGTTCATATTTCCGATGGCG	1579 bp	1384 bp	GT (tag)	This study
PBANKA_1004700-CT-R	TTACTAGTTTAGCTTAAATGCGC		1516 bp	GT (tag)	This study
PBANKA_1212100-3'-F	TTTTACTATTTATTATTACGCGTTGC	967 bp		GT (tag)	This study
PBANKA_1212100-3'-R	AGTGTCTAAAGGAAGTAATGTAGC		1403 bp	GT (tag)	This study
PBANKA_1212100-CT-F	GCTAAATGGAATGCTGATTCG	1246 bp	1070 bp	GT (tag)	This study
PBANKA_1212100-CT-R	ATACGCAATATATGAAACCCGG			GT (tag)	This study
PBANKA_1349200-CT-F	TTTAAGCTATGTTTTGCACGC	1366 bp	1423 bp	GT (tag)	This study
PBANKA_1349200-CT-R	TAAATAATGGTGCAATCATAAGTATCC		1437 bp	GT (tag)	This study
PBANKA_1350500-CT-F	ATATTCCTGGGTAATGGTTTTATTCC	2280 bp	2172 bp	GT (tag)	This study
PBANKA_1350500-CT-R	CCATAAGTACAAGCTATTTGAGC		1509 bp	GT (tag)	This study
PBANKA_1441700-CT-F	CCACAAAGAGAAGCAAATAATGG	1469 bp	1412 bp	GT (tag)	This study
PBANKA_1441700-CT-R	CAAAAATATGGAAATCACTTACAGGG		1383 bp	GT (tag)	This study
PBANKA_0826700-5'-F-SacII	TTTTTTCCGCGGTTATTTGTTATTTTTTATGAATTTGCGG	1003 bp		TV (KO)	This study
PBANKA_0826700-5'-R-PvuII	AACTATCAGCTGTATTAAAAAATACAAATAACGACCCACC			TV (KO)	This study
PBANKA_0826700-3'-F-XhoI	AATAATCTCGAGAATAACCATGAAATAAGCAAACAGG	872 bp		TV (KO)	This study
PBANKA_0826700-3'-R-KpnI	TTAAAAGGTACCCAACATCACATACGTGAAGAAG			TV (KO)	This study
PBANKA_0919100-5'-F-SacII	AATATTCCGCGGTTAATTAACCTAAAACGTTTCCGG	871 bp		TV (KO)	This study
PBANKA_0919100-5'-R-EcoRI	TTATTAGAATTCCGCCATTATTTTGAATGCACC			TV (KO)	This study

Supplementary Table S1. (continued)

PBANKA_0919100-3'-F-XhoI	TTTTTTCTCGAGCTGTACATTAGTATCATTTCATTATTCC	984 bp		TV (KO)	This study
PBANKA_0919100-3'-R-KpnI	AATAATGGTACCGGTTAAGTATGGAATGTCTTCAGC			TV (KO)	This study
PBANKA_1349200-5'-F-SacII	ATAAAACCGCGGAAATATTGTCATCAGCATTGTTGG	808 bp		TV (KO)	This study
PBANKA_1349200-5'-R-PvuII	TTTTTTCAGCTGTATAATTTGGTGATTTCTTTTTGCG			TV (KO)	This study
PBANKA_1349200-3'-F-XhoI	TTAATGCTCGAGTGAAAGGATACTTATGATTGCACC	1001 bp		TV (KO)	This study
PBANKA_1349200-3'-R-KpnI	TTTTTTGGTACCTTTTATTTTTGGTGAATTCCCTAGC			TV (KO)	This study
PBANKA_1350500-5'-F-SacII	TATACTCCGCGGTCATAGGAAAGTTATTTCATTTATTATCC	847 bp		TV (KO)	This study
PBANKA_1350500-5'-R-PvuII	AAAAACCAGCTGAAAGTGAGAATGAGCTTTAAAACC			TV (KO)	This study
PBANKA_1350500-3'-F-XhoI	TTTTTACTCGAGGCTCAAATAGCTTGTACTTATGG	837 bp		TV (KO)	This study
PBANKA_1350500-3'-R-KpnI	AATAATGGTACCGGTAAGTGAAGTTAATAGTAGAATCCC			TV (KO)	This study
PBANKA_1441700-5'-F-SacII	TTTTTACCGCGGAGGCTGCTTTCTTTAATGTCC	716 bp		TV (KO)	This study
PBANKA_1441700-5'-R-EcoRI	AATTTTGAATTCCCCTCTCTAATATCCTCATCG			TV (KO)	This study
PBANKA_1441700-3'-F-XhoI	ATTATACTCGAGAGATTGTTTTTTATGTGTTATTTGCG	1020 bp		TV (KO)	This study
PBANKA_1441700-3'-R-KpnI	AATAATGGTACCTAAATGTGGACAGCAACAAAGG			TV (KO)	This study
PBANKA_0826700-5'-F	ACTCTGTGCATAGAATAATGCC	1575 bp	1205 bp	GT (KO)	This study
PBANKA_0826700-5'-R	GGGAATCTTTATTATTGTTTGAGGC			GT (KO)	This study
PBANKA_0826700-3'-F	TTTAAACTGGCCATTCATTCTCC	1591 bp		GT (KO)	This study
PBANKA_0826700-3'-R	TTACTAAAATATGGAAAGACATTTATGC		1844 bp	GT (KO)	This study
PBANKA_0919100-5'-F	GTTTTTATTTAGTCCCTATTTGTATATGC	1426 bp	1233 bp	GT (KO)	This study
PBANKA_0919100-5'-R	ATGGATAAGAAAATTGCTAACGC			GT (KO)	This study
PBANKA_0919100-3'-F	CCTACCTCATGAAAATATGAACCC	1133 bp		GT (KO)	This study
PBANKA_0919100-3'-R	TTGTATTTCGTGTACCATTTATATAGG		1637 bp	GT (KO)	This study
PBANKA_1349200-5'-F	TATATAAGCGGCTAAAAAAAGGG	1492 bp	1220 bp	GT (KO)	This study
PBANKA_1349200-5'-R	AATATAAGGCTAATACTACTAAAGGG			GT (KO)	This study
PBANKA_1349200-3'-F	CCGATAATGATAAAATGTACATGGC	1258 bp		GT (KO)	This study
PBANKA_1349200-3'-R	GTTTTATATGTCCGCATGTTGC		1801 bp	GT (KO)	This study
PBANKA_1350500-5'-F	TATCCTAATTTGAATGTATAAGAATATGG	1534 bp	1269 bp	GT (KO)	This study
PBANKA_1350500-5'-R	AATGTGGCTGTACAAGAGGG			GT (KO)	This study
PBANKA_1350500-3'-F	TGATGGAAATGTAAACAATGGTGG	1229 bp		GT (KO)	This study
PBANKA_1350500-3'-R	TGTAATACATAAATGGCACGTTCC		1482 bp	GT (KO)	This study
PBANKA_1441700-5'-F	TTATTTGTTTGCATTTGAAATGAGC	1077 bp	1145 bp	GT (KO)	This study
PBANKA_1441700-5'-R	AGTGGTTTTTCGAAGGAATACG			GT (KO)	This study
PBANKA_1441700-3'-F	AAAACAAATAGAACGTATAATTATAGGC	1121 bp		GT (KO)	This study
PBANKA_1441700-3'-R	TCTTGATTGTTTTTTAACCTTTAAGC		1682 bp	GT (KO)	This study
GFP-PV-F	GCATTTGTTTTAGTATTATTTGTATCCC	807 bp		TV (ER)	This study
GFP-PV-NSLD*-R-BamHI	TTTTCTGGATCCTTATAAATCTGAATTGGTACCGGCGCCTTTGTATAGTTCATC	807 bp		TV (ER)	This study
GFP-PV-IIGN*-R-BamHI	ATTTTTGGATCCTTAATTACCTATTATGGTACCGGCGCCTTTGTATAGTTCATC	807 bp		TV (ER)	This study
GFP-PV-EKKK*-R-BamHI	ACATTTGGATCCTTACTTCTTCTTTTCGGTACCGGCGCCTTTGTATAGTTCATC	807 bp		TV (ER)	This study
GFP-PV-NLDN*-R-BamHI	ATCTTTGGATCCTTAATTATCTAAATTGGTACCGGCGCCTTTGTATAGTTCATC	807 bp		TV (ER)	This study
GFP-PV-LNSM*-R-BamHI	TAATTTGGATCCTTACATACTATTTAAGGTACCGGCGCCTTTGTATAGTTCATC	807 bp		TV (ER)	This study
GFP-PV-TFCD*-R-BamHI	ATAGTTGGATCCTTAATCACAGAATGTGGTACCGGCGCCTTTGTATAGTTCATC	807 bp		TV (ER)	This study
5'HSP70rev	CAATTTGTTGTACATAAAATAGGCAG			GT	Kenthirapalan et al., 2012
5'DHFRrev	ATGAAATACCGCTCCATTTTTCC			GT	Kenthirapalan et al., 2012
mCherryRev	CCCTCCATGTGAACCTTGAAG			GT	Haussig et al., 2011

[a] Sizes of the PCR products of forward and reverse primers on wild type (WT) genomic DNA.

[b] Sizes of the respective integration-specific PCR products (INT); forward 5' gene-specific primers combined with 5'HSP70rev, carboxy-terminal tagging-specific forward primers combined with mCherryRev, and reverse 3' gene-specific primers combined with 5'DHFRrev (double homologous recombination) or M13F (single homologous recombination).

[c] Primers used for construction of Transfection Vectors (TV) or for GenoTyping (GT) of either knockout mutants (KO), tagging mutants (tag) or mutants expressing ER retrieval testing constructs (ER).

SUPPLEMENTARY REFERENCES

- Haussig, J. M., Matuschewski, K. & Kooij, T. W. A. Inactivation of a *Plasmodium* apicoplast protein attenuates formation of liver merozoites. *Mol. Microbiol.* **81,** 1511–1525 (2011).
- Kenthirapalan, S., Waters, A. P., Matuschewski, K. & Kooij, T. W. A. Flow cytometry-assisted rapid isolation of recombinant *Plasmodium berghei* parasites exemplified by functional analysis of aquaglyceroporin. *Int. J. Parasitol.* **42,** 1185–1192 (2012).
